# Clinical experience of using integrated whole genome and transcriptome sequencing as a framework for pediatric and adolescent acute myeloid leukemia diagnosis and risk assessment

**DOI:** 10.1038/s41375-025-02774-5

**Published:** 2025-10-07

**Authors:** Rebecca K. Voss, Victor B. Pastor Loyola, Maria F. Cardenas, Priya Kumar, Jamie L. Maciaszek, Maria Namwanje, Jing Ma, Jennifer L. Neary, Meiling Jin, Masayuki Umeda, Mark R. Wilkinson, Debbie Payne-Turner, Mohammad K. Eldomery, Jingqun Ma, Jiali Gu, Jim Dalton, Samantha Melton, Yen-Chun Liu, Scott Foy, Michael Rusch, David A. Wheeler, Jinghui Zhang, Kim E. Nichols, Seth E. Karol, Hiroto Inaba, Raul Ribeiro, Jeffrey E. Rubnitz, Jeffery M. Klco, Lu Wang

**Affiliations:** 1 Department of Pathology, St. Jude Children’s Research Hospital, Memphis, TN USA; 2 Department of Computational Biology, St. Jude Children’s Research Hospital, Memphis, TN USA; 3 Department of Oncology, St. Jude Children’s Research Hospital, Memphis, TN USA

**Keywords:** Genetic testing, Cancer genomics

## Abstract

Pediatric acute myeloid leukemia (AML) exhibits distinct genetic characteristics, including unique driver alterations and mutations with prognostic and therapeutic implications. Cytogenetics study, along with Next Generation Sequencing (NGS) panel testing, have long been the standard for molecular diagnosis of AML. While these approaches enable diagnosis and prognosis determination in most cases, they have limitations—particularly in detecting emerging rare, recurrent genetic abnormalities. In this study, we systematically reviewed our real-time clinical experience with the diagnostic workup of pediatric AML using an integrated whole genome and whole transcriptome sequencing (iWGS-WTS) approach and compared the test results obtained from various methodologies, including whole genome sequencing (WGS), whole exome sequencing (WES), whole transcriptome sequencing (WTS), iWGS-WTS, cytogenetics, and targeted panel NGS. Our findings demonstrate that the iWGS-WTS approach improves the identification of clinically relevant genetic alterations, enhancing precise disease classification and risk assessment. Additionally, the iWGS-WTS approach streamlines sample acquisition and reduces testing redundancy, positioning it as a practical and superior alternative to traditional diagnostic methods in pediatric AML management.

## Introduction

Acute myeloid leukemia (AML) comprises a heterogeneous group of genetically distinct disorders. Over the past decade, next-generation sequencing (NGS) has uncovered the molecular landscape of AML, highlighting recurrent genetic alterations and their interactions [1–4]. These insights, alongside efforts to understand myeloid cell transformation, have shaped the molecular classification of AML, leading to the inclusion of additional genetically defined subtypes in the updated WHO classification [5]. Although AML accounts for only 20% of pediatric acute leukemia cases, it is the leading cause of childhood leukemia mortality [6]. While sharing features with adult AML, pediatric AML displays distinct genetic characteristics [3, 7]. The continued discovery of rare but recurrent genetic abnormalities and their impact on patient outcomes underscores the need for high-throughput molecular diagnostics and unbiased detection strategies.

In 2016, St. Jude Children’s Research Hospital (SJCRH) implemented comprehensive clinical genomics testing for leukemia molecular diagnosis, combining WGS, WES, and WTS. This study systematically reviewed the workflow and results from real-time molecular diagnostic evaluations in pediatric AML patients, focusing on the performance of WGS and WTS compared to other molecular and cytogenetic tests. The findings demonstrated that an integrated WGS-WTS (iWGS-WTS) approach is a robust, comprehensive, and efficient tool for pediatric AML diagnosis, classification, and risk assessment.

## Materials and methods

One hundred and fifty-three pediatric and adolescent AML patients admitted to SJCRH and/or enrolled in SJCRH-sponsored clinical trials between 2016 and 2021 were included in the study cohort (Supplementary Table S1). Informed consent for clinical genomics and research was obtained from all patients. Tumor and matched germline samples were collected from each patient for paired tumor-normal clinical genomics testing as part of the diagnostic workup in our Clinical Laboratory Improvement Amendments (CLIA)-certified Molecular Pathology clinical laboratory. Bone marrow aspirate or peripheral blood samples with documented blast counts were used as tumor samples. Skin biopsy samples were collected as germline comparators; other tumor-free samples were used for patients when skin biopsy samples were not available. All patients underwent real-time genomic and transcriptomic profiling of their tumor samples using WGS, WES, and WTS, with WES performed alongside WGS to internally assess the detection of single-nucleotide and small insertion-deletion variants (SNV/Indel). Material from 136 of 153 patients was subjected to cytogenetics evaluation in the Clinical Cytogenetics laboratory at SJCRH as part of the traditional diagnostic workup. Results from a targeted NGS panel test obtained from the same DNA sample used for WGS/WES were available for 39 patients. Details on sample process of each testing platform, data analysis, as well as NGS data annotation, variant process and curation, are described in Supplementary Information and Supplementary Fig. S1. This systematic review study was approved by the Institutional Review Board (IRB) at SJCRH.

## Results

### Characteristics of the cohort and sequencing performance

The study cohort of 153 patients had a nearly equal sex distribution (74 male, 79 female) and a median age at diagnosis of 11 years (range 1 week–22 years). Samples from the first available timepoint were analyzed, comprising 13 AML post-cytotoxic therapy, 27 relapsed AML cases, of which the initial diagnosis and treatment were conducted outside SJCRH, and 113 cases with de novo AML. Median bone marrow blast count of analyzed samples was 63% (range 5–96%) with blast content exceeding 20% in all but four cases (Supplementary Table S1 for details). Sequencing data quality metrics across the study cohort were evaluated and presented in Supplementary Table S2 and Supplementary Figs. S2 and S3.

### Comprehensive tumor genomic and transcriptomic profiling and comparison between NGS platforms

#### Detection of SNVs/indels and comparison of iWGS-WTS to both WES and targeted NGS

**Fig. 1:**
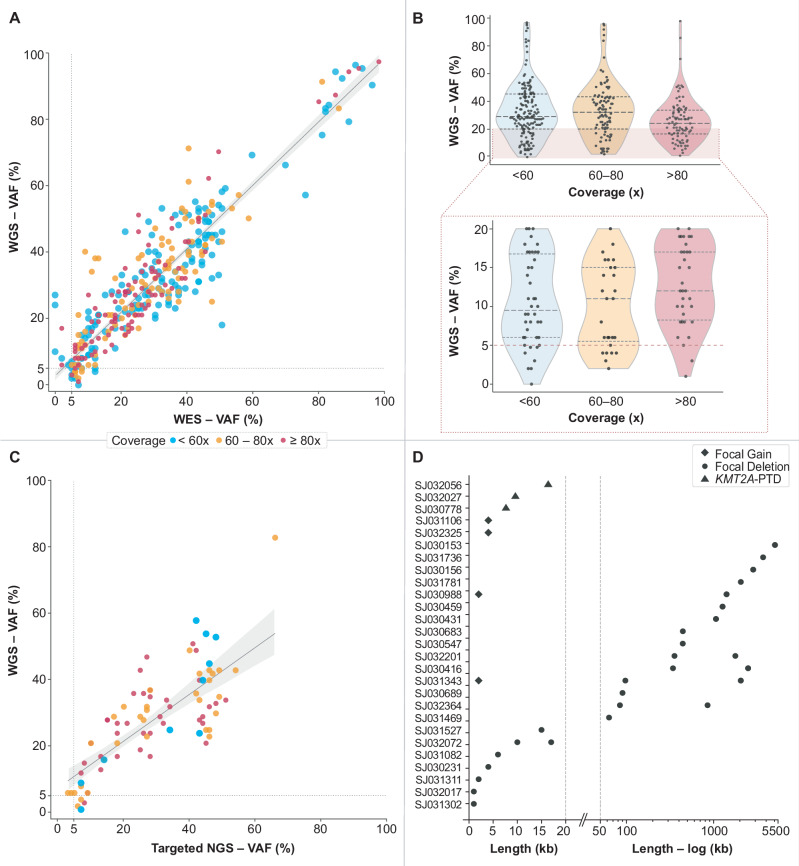
Detection of SNVs/indels and focal CNVs. **A** Comparison of Whole Genome Sequencing (WGS) and Whole Exome Sequencing (WES) in the detection of SNVs and Indels. Variant allele frequency (VAF) for WES and WGS is shown on the *x* and *y* axes, respectively. High concordance in VAF estimates among the variants considered reportable by both WGS and WES was observed: correlation coefficient (*r*) = 0.9 (slope = 0.88). **B** Violin plot showing the distribution of variants detected across different WGS coverage levels per sample. The *x*-axis represents the WGS coverage, divided into three groups: <60×, 60–80×, and >80×. The *y*-axis represents the VAF in WGS of each variant. **Upper section** includes all SNVs/indels shown in A); **lower section** focuses on variants of ≤20% VAF by WGS. The dashed lines within the violins represent the quartiles (25th, 50th [median], and 75th percentiles). **C** Comparison of WGS and Targeted NGS in the detection of SNVs and indels. VAF for Targeted NGS and WGS are shown on the *x* and *y* axes, respectively. Good concordance in VAF estimates between WGS and the Targeted NGS with a correlation coefficient (*r*) of 0.77 (slope = 0.82), was observed. **D** Focal CNVs detected by WGS are presented according to size (in kb) on the *x*-axis, with a linear scale from 0 to 50 kb and a logarithmic scale from 50 kb to 5500 kb. Abbreviations: CNV copy number variant, kb kilobase, NGS next generation sequencing, PTD partial tandem duplication, SNV single nucleotide variant, Indel small insertion-deletion variant.

Using the iWGS-WTS approach, a total of 330 somatic pathogenic or likely pathogenic (P/LP) SNV/Indels in 135/153 patients were considered valid for reporting (Supplementary Table S3), including 327 variants with WGS VAF ≥5% and three AML-driver variants (each identified in individual cases) with VAF <5% in WGS but exceeding 25% in WTS (Supplementary Table S3). Next, we compared the performance of iWGS-WTS and WES in detecting SNVs/indels with VAFs ≥5%. Of the 327 variants, 318 were also detected by WES with VAF ≥5%. Among the nine additional variants not called by WES, the majority were complex indels, and seven were confirmed through manual review of WES BAM files with VAFs ranging from 0.3% to 4.9%. Conversely, 15 variants detected by WES with VAFs ranging from 5–12.5% (average 6.8%) did not meet the established reporting criteria for iWGS-WTS. Manual review of WGS BAM files showed all but one (average VAF: 3.1%; range: 1.1–4.8%). Together, among the 342 variants, ~96% with VAF ≥5% (range: 5 to 97%) were considered reportable by both iWGS-WTS and WES, with high concordance in VAF estimates between WGS and WES (Fig. 1A). After accounting for WGS coverage, we found no significant association between low VAF and low coverage (Fig. 1B).

The iWGS-WTS testing for SNVs/indels was further assessed in 39 cases, of which targeted NGS DNA-seq (75-gene panel) results were available (Supplementary Table S3). Of the 74 P/LP variants with VAF ≥5% identified by the panel test, 70 were also valid by iWGS-WTS. One additional variant detected by iWGS-WTS (VAF > 5%) was reported at VAF of 4% in the panel. High concordance in VAF estimates between WGS and targeted NGS was observed (Fig. 1C). Five additional variants with estimated VAF between 3 and 5% reported by the panel test were observed in WGS BAM files upon manual review, but did not meet the established reporting criteria for iWGS-WTS.

In addition to genomic profiling of somatic mutations, 220 genes (Supplementary Table S4A) predisposing to cancer and/or bone marrow failure that are clinically validated for germline molecular diagnosis at SJCRH were evaluated in each patient’s germline sample. A total of 20 P/LP SNVs/indels were identified in 19 patients (~12%) by both WGS and WES with comparable VAF (Supplementary Table S4B).

#### Detection of FLT3-intragenic tandem duplications (ITD), focal and large-scale CNVs

Of the 153 patients analyzed, 18 were found to harbor a total of 28 *FLT3*-ITDs. Fifteen of 28 showed strong evidence in both WGS and WTS, while two *FLT3*-ITDs seen in one patient had strong evidence in WGS but lacked sufficient coverage and supporting reads in WTS. Conversely, 10 *FLT3*-ITDs demonstrated strong evidence in WTS but weak or no evidence in WGS, making them borderline findings. The remaining one exhibited weak evidence in both WGS and WTS. All cases were confirmed by PCR-based fragment analysis (Supplementary Table S5A). Additionally, PCR identified one additional subclonal *FLT3*-ITD (ratio of *FLT3*-ITD to wild-type: 0.02) in SJ030286 that was not detected by WTS or WGS.

Furthermore, WGS revealed 42 P/LP focal CNVs (<5 Mb) in 24 patients, including 15 alterations smaller than 50 kb (12 ≤ 10 kb), presenting as intragenic/exonic CNVs leading to truncation of the functional protein (Fig. 1D and Supplementary Table S5B). Among the alterations smaller than 50 kb, the recurrent findings were deletions in *CBL* (*n* = 4) affecting exons 8 and/or 9 (Supplementary Fig. 4A) and *KMT2A* partial tandem duplication (*KMT2A*-PTD, *n* = 3). All *KMT2A*-PTD alterations were also supported by WTS, which identified in-frame fusions of exons 7 or 8 upstream of a 5’ exon (exons 2, 3, or 4) (Supplementary Fig. 4B).

Genome-wide large-scale copy number variants (LS-CNV, ≥5 Mb) and copy-neutral loss of heterozygosity (cnLOH, ≥10 Mb) detected by WGS analysis are presented in Supplementary Table S6. In general, 163 LS-CNV events were identified in 83 patients. Additionally, 18 patients had large-scale cnLOH identified by WGS.

#### Detection of AML-associated gene fusions/rearrangements and comparison of WGS and WTS

**Fig. 2:**
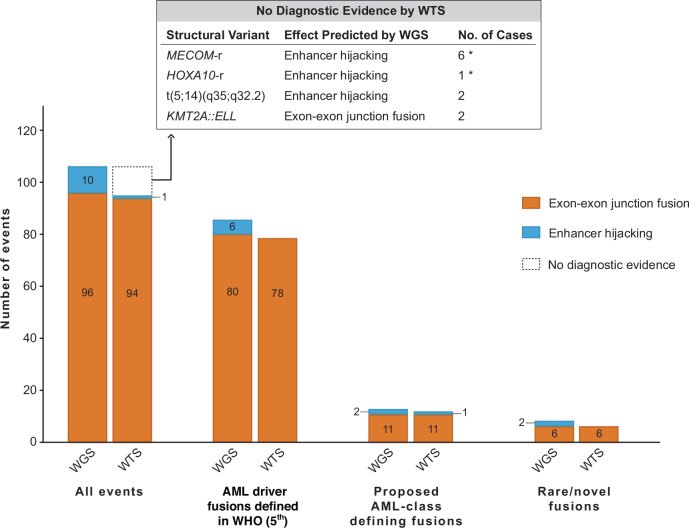
Comparison of WGS and WTS in detecting AML-associated oncogenic or likely oncogenic gene fusions and chromosomal translocations. Paired bars represent the number of events detected by WGS or WTS. “All events” includes all 106 AML-associated oncogenic/likely oncogenic fusions/chromosomal translocations revealed by WGS and their detection by WTS. “All Events” are further categorized into three groups for comparison: AML driver fusions defined in WHO (5^th^ edition), proposed AML class-defining fusions, and rare/novel fusions. Alterations with no/insufficient diagnostic evidence of fusion transcripts by WTS are summarized in the table on top of the bar chart. The asterisk (*) indicates alterations with elevated oncogene expression levels in WTS, supporting the enhancer hijacking structural alterations identified by WGS. Conversely, for the two cases with t(5;14)(q35;q32.2) revealed by WGS, the proposed enhancer hijacking mechanism could not be validated by gene expression data from WTS (see Supplementary Fig. S7). **Abbreviations**: No. number, -r -rearrangement, WGS whole genome sequencing, WHO world health organization, WTS whole transcriptome sequencing.

WGS analysis for structural variants suggested 106 AML-associated oncogenic or likely oncogenic fusions in 105 cases (SJ031259 harbored both *CBFB::MYH11* and a *CNTRL::KIT* fusion), of which 96 were predicted to produce fusion oncogenes (in-frame exon-exon junction fusions) and 10 were suspected to be enhancer-hijacking structural alterations that activate adjacent oncogenes (Fig. 2). WTS analysis alone diagnosed 94/96 (98%) P/LP WGS-detected fusion oncogenes, with no false positive findings (Supplementary Table S7) according to our established diagnostic criteria. In the remaining two cases, SJ031554 and SJ031359, WGS suggested *KMT2A::ELL* fusions, which were further confirmed by targeted RNA sequencing (Supplementary Fig. 5), despite no diagnostic evidence of the fusion in WTS.

The 10 cases with suspected enhancer hijacking fusions detected by WGS were predicted to dysregulate *MECOM* in six cases, *HOXA* genes in two (SJ030410 and SJ032072), and *BCL11B* (or *TLX3*) in two (SJ030153 and SJ030431) based on WGS analysis. Among them, only one case had a chimeric fusion transcript revealed by WTS: a fusion transcript for *CDK6::HOXA13* in SJ030410, along with overexpression of *HOXA13* (Supplementary Fig. S6A, B). In this case, WGS identified a rearrangement that juxtaposed the enhancer RNA *CDK6*-AS1 [8] on 7p21.2 to the 3’-UTR of *HOXA13*. Across the six *MECOM*-rearranged cases, WGS showed breakpoints on 3q26.2, ranging from 3.8 kb to 300 kb upstream and from 78 kb to 146 kb downstream of *MECOM* (NM_001105077.3, Supplementary Fig. S6C). These regions adjacent to *MECOM* were fused to the *GATA2* locus at 3q21.3 (*n* = 2), the *CDK6* locus at 7q21.2 (*n* = 3), and an intergenic region at 2p21 (*n* = 1). Although no reads of chimeric transcripts were present in WTS, high expression of *MECOM* was observed in all six cases (Supplementary Fig. S6D), which aligns with the mechanism of enhancer hijacking previously described [9]. Notably, *MECOM*-r cases associated with *CDK6* showed fusion junctions in intron 2 or 3 of *CDK6* (Supplementary Fig. S6E), suggesting these leukemias are utilizing alternative enhancers within the *CDK6* locus other than *CDK6-AS1*. Similar evidence supporting enhancer-hijacking chromosomal rearrangement and *HOXA10* activation was observed in SJ032072.

SJ030153 and SJ030431, classified as FAB-M0 and M1, respectively, exhibited a translocation t(5;14)(q35;q32) by WGS (Supplementary Fig. S7A). Since 14q32 rearrangements deregulating *BCL11B* define a distinct subtype of T-/myeloid-MPAL and immature myeloid leukemia, we assessed *BCL11B* expression. Both cases showed *BCL11B* levels comparable to non-*BCL11B* AML cases and dramatically lower than the *BCL11B*-rearranged AML subtype. Genomic profiling of both cases revealed features resembling T-ALL with t(5;14)(q35;q32) [10–12], including alterations in *PHF6* and focal deletions of *CDKN2A/B*. However, WTS showed no overexpression of *TLX3* or *NKX2-5*, contradicting the predicted enhancer hijacking mechanism (Supplementary Fig. S7B, C). Together, the different case scenarios described above underscore the importance of an iWGS-WTS approach for diagnosing enhancer hijacking structural alterations.

#### Expression data from WTS improves interpretation and molecular classification of variants

Further on, combined analyses of sequence variants, expression of involved genes (or alleles), and global gene expression profiling through WTS offer critical insights into the biology and clinical significance of genetic findings, especially when novel alterations are identified. For instance, the *HSPA8::PRDM16* fusion identified in SJ031206 was deemed biologically similar to other *PRDM16* rearrangements reported in AML, based on its fusion structure and expression profile [13, 14] (Supplementary Fig. S8A–C). In another case, SJ032178, WGS and WTS identified an acquired frameshift variant in the N-terminal transactivation domain of *CEBPA*, with a VAF of 35% in genomic DNA and 97% in WTS, respectively, indicating exclusive expression of the mutant allele in tumor. This case also clustered with bi*CEBPA*/smbZIP-*CEBPA* AML [5] based on global gene expression profiling (GEP) (Supplementary Fig. S9). Together, WTS findings strongly support diagnosing this patient’s leukemia as AML with *CEBPA* mutation. Additional example case scenarios presented in Supplementary Fig. S10 further highlight the value of GEP in AML molecular diagnostics.

### Comparing the iWGS-WTS approach to conventional cytogenetics

#### Detection of driver gene fusions

We next compared results from iWGS-WTS with G-banded karyotyping for the detection of chromosome translocations/gene fusions in 136 cases tested with conventional cytogenetics. Among these cases, 90 had known or potential AML-driver gene fusions established by iWGS-WTS, of which 71 (~79%) were detected by cytogenetics (Table 1 and Supplementary Table S8A, B). Discordant findings consisted mainly of rare AML fusions discovered in recent years by advanced sequencing technologies and known to be cytogenetically cryptic. Additionally, three cases with traditional AML-defining gene fusions routinely evaluated by cytogenetic analysis, *CBFB::MYH11* (SJ032253), *RBM15::MRTFA* (SJ030209), and *KMT2A::MLLT10* (SJ030361), were only identified by iWGS-WTS (Table 1). None of the cytogenetically positive AML-associated chromosome rearrangements were missed by iWGS-WTS.

**Table 1:** Comparison between iWGS-WTS and conventional cytogenetics in the detection of AML-defining gene fusions/rearrangements.

Gene rearrangement/fusion	iWGS-WTS and cytogenetics	iWGS-WTS only	Total	Missed by cytogenetics
*RUNX1::RUNX1T1*	23		23	0%
*CBFB::MYH11*	10	1^c^	11	9%
*KMT2A::MLLT3*	9		9	0%
*KMT2A::MLLT10*	6^b^	1^c^	7	14%
*KMT2A::ELL*	4	1	5	20%
*KMT2A::MLLT4*	2		2	0%
*KMT2A::MLLT1*	1		1	0%
*KMT2A::MLLT6*	2		2	0%
*KMT2A::MLLT11*	1		1	0%
*PML::RARA*	2		2	0%
*TBL1XR1::RARB*	1		1	0%
^a^*NUP98::KDM5A*		1	1	100%
^a^*NUP98::NSD1*		1	1	100%
*MECOM-r*	2	4	6	67%
*DEK::NUP214*	1		1	0%
*RBM15::MRTFA*		1	1	100%
*PICALM::MLLT10*	2		2	0%
*KAT6A::CREBBP*	1		1	0%
*FUS::ERG*	1		1	0%
*RUNX1::CBFA2T3/2*	2	1	3	33%
^a^*CBFA2T3::GLIS2*		1	1	100%
*MYB::GATA1*		1	1	100%
*CDK6::HOXA13*		1	1	100%
*TEC::MLLT10*		1	1	100%
*HSPA8::PRDM16*		1	1	100%
*RUNX1::EVX1*	1		1	0%
*RUNX1::POU2F2*		1	1	100%
*RUNX1::ZEB2*		1	1	100%
*RUNX1::USP42*		1	1	100%
Total	71	19	90	21%

FISH (Fluorescence in situ hybridization) analysis for *KMT2A* rearrangement, *CBFB* rearrangement and *RUNX1::RUNX1T1* fusion were available in a subset of cases (See Supplementary Table S1).

iWGS-WTS integrated WGS and WTS.

^a^Known cytogenetically cryptic gene fusions.

^b^For cytogenetic testing in these 6 cases, *KMT2A::MLLT10* fusions were defined by metaphase FISH using *KMT2A* break-apart FISH probe.

^c^Break-apart FISH analysis for *CBFB* and *KMT2A* was performed on SJ032253 (*CBFB::MYH11*) and SJ030361 (*KMT2A::MLLT10*), respectively, but revealed no evidence of gene rearrangements, suggesting that a cryptic insertion mechanism may underline the formation of gene fusions in both cases.

#### Solving complex genomes and identifying drivers in cases with complex karyotypes

We also assessed the ability of iWGS-WTS to resolve complex genomes and uncover critical pathogenic alterations in a series of nine cases where conventional cytogenetics showed complex karyotype without further diagnostic information. Indeed, iWGS-WTS identified AML-defining genetic alterations in seven of these nine cases (1 *KMT2A::ELL*, 1 *RBM15::MRTFA*, 1 *NUP98::KDM5A*, 1 *NPM1* mutation, and biallelic alterations of *TP53* in 3 cases), and revealed a *CDK6::HOXA13* fusion and a *TEC::MLLT10* (rarely reported in-frame fusion) in the remaining two cases (SJ030410 and SJ030773), respectively (Supplementary Table S8B). Additionally, although genome-wide findings from WGS and conventional cytogenetics were largely consistent, WGS illustrated the genomic complexity with granular detail and an accuracy superior to karyotype analysis. See example cases in Supplementary Fig. S11A, B.

#### Detection of large-scale CNVs and comparison between WGS and conventional cytogenetics

We next evaluated the concordance between WGS and conventional cytogenetics in detecting LS-CNVs that are theoretically visible by cytogenetics at a band level of 400 bands per haploid (bphs). This banding resolution allows discrimination of copy-number changes of ~9 Mb or higher [15]. Of the 136 cases for which karyotyping was performed, 126 with non-complex karyotype were used for a head-to-head comparison study (Supplementary Table S8A).

The overall agreement in LS-CNV findings between WGS and conventional cytogenetics was ~77% (80/104) (Supplementary Fig. S12), including abnormal chromosomes described as unbalanced structural alterations (“add” or “del”) or marker chromosome (“mar”) by conventional cytogenetics and further clarified by WGS in five cases (SJ031527, SJ031601, SJ031719, SJ032364 and SJ032526). The chromosome abnormalities of these five cases are described in Supplementary Table S8A (see “Comments”) and illustrated in CIRCOS plots, which integrated the visualization of genome-wide SVs and CNVs revealed by WGS (Supplementary Fig. S13A–E). Both testing platforms detected certain aberrations that were not identified by the other. Eight of nine CNVs identified only by conventional cytogenetics were subclonal findings observed in <50% of analyzed metaphases, and none defined myelodysplasia-related AML (AML-MR) [5]. The remaining CNV, a trisomy 17 in SJ030079, was likely misinterpreted in karyotype due to poor-quality metaphases. The WGS performance for detecting low allele fraction LS-CNVs was also evaluated through a series of dilution experiments using COLO829 tumor cell line and its matched normal COLO829L at tumor DNA purities of 100%, 40%, and 30% (Supplementary Information). The detection of LS-CNV remained reliable at 40% but declined at lower purities (Supplementary Fig. S14), which is in line with what we observed through the comparison study with conventional cytogenetics.

Among the 15 events identified by WGS only, four were relatively small CNVs (10–20 Mb), which could be challenging to detect using conventional cancer cytogenetics due to metaphase quality and/or resolution limitations of G-banding; the remaining were mainly CNVs associated with unbalanced chromosomal translocations, which were missed by conventional cytogenetics due to banding similarity of the chromosome segments involved. It should be noted that the agreement between WGS and conventional cytogenetics to call AML-MR cytogenetic abnormalities [5] throughout the cohort was ~90% (28/31) (Table 2). All three discordant findings were segmental deletions missed by conventional cytogenetics, including two cases with deletions in 7q and one deletion in 11q.

**Table 2:** Comparison of WGS and conventional cytogenetics in the detection of AML-MR defining cytogenetic alterations.

AML-MR defining	WGS and cytogenetics	WGS only	Total	Missed by cytogenetics
5q-	2		2	0%
mono 7 / 7q-	13	2	15	13%
11q-	1	1	2	50%
12p-	1		1	0%
17p-	1		1	0%
i[17q]	1		1	0%
Complex karyotype	9		9	0%
Total AML-MR defining	28	3	31	10%

AML-MR acute myeloid leukemia, myelodysplasia-related, mono 7 monosomy of chromosome 7, i[17q] isochromosome 17q.

### Molecular diagnosis and classification through integrated genomic and transcriptomic profiling

#### Molecular subtypes defined by integrated genomic and transcriptomic profiling

**Fig. 3:**
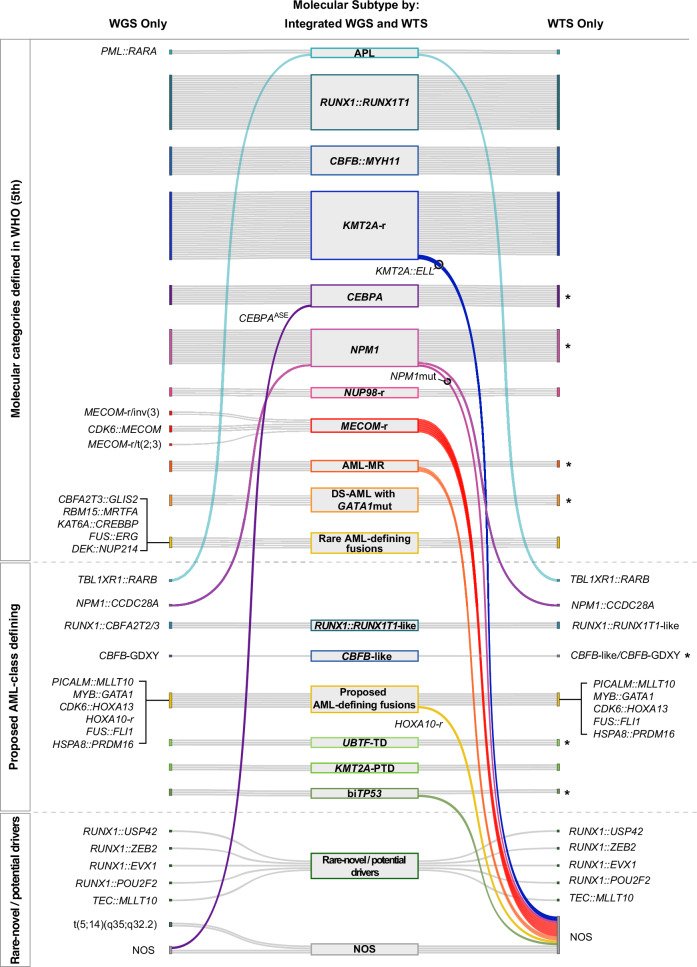
Molecular classification of cases in the study cohort. Comparison of tumor profiling approaches: WGS, WTS, and integrated WGS and WTS (iWGS-WTS), for the molecular classification of AML. Detection of AML genetic drivers by WGS only is shown on the left, WTS only on the right, and the final molecular driver/subtype determined by iWGS-WTS is presented in the center. If the molecular driver identified by WGS or WTS alone aligns with the final subtype determined by iWGS-WTS, the specific genetic alteration is not redundantly listed under WGS or WTS. The asterisk (*) indicates that establishing a reliable pipeline is essential for accurately diagnosing SNVs/indels using WTS alone. **Abbreviations**: AML-MR acute myeloid leukemia, myelodysplasia-related, biTP53 biallelic *TP53* alterations, *CBFB*-GDXY recurrent in-frame insertion mutations in *CBFB*, leading to a GDXY amino acid sequence change at position D87, *CEBPA*-ASE *CEBPA*-allele specific expression, DS-AML down syndrome related AML, mut mutation, NOS not otherwise specified (no known/proposed or predicted AML-defining genetic alteration detected), -r -rearrangement, SNV single nucleotide variant, WGS whole genome sequencing, WTS whole transcriptome sequencing.

The molecular subtype of each case was determined based on the comprehensive and real-time genomic and transcriptomic profile by iWGS-WTS. When comparing the molecular classification by WGS alone, WTS alone and iWGS-WTS, we observed the highest diagnostic detection rate and precision with iWGS-WTS (97.4%, Fig. 3). Of the 148 non-Down syndrome patients, 86 were characterized by AML class-defining gene fusions [5], 26 by AML-class defining mutations [5], 21 defined by newly proposed/recognized AML class-defining variants [7, 14, 16–27], and five cases showed AML-MR defining gene mutations and/or cytogenetic abnormalities in the absence of any other known or potential genetic drivers of AML (Supplementary Table S9). Of the other 10 cases, novel or rare in-frame gene fusions previously reported in hematological malignancy case reports in literature [28–32] were identified in six, while the genetic driver for the last four cases remained unclear. Pathogenic *GATA1* variants were identified in all five patients with Down syndrome.

We next evaluated the effectiveness of standard AML diagnostic tests, conventional cytogenetics with routine AML FISH (CC + AML-FISH), and the commonly used NGS panel (FoundationOne Heme) for molecular diagnosis and classification in the study cohort. We estimated that ~58% of cases in this cohort could be accurately diagnosed and classified using CC + AML-FISH, and 86% by FoundationOne Heme test (based on the genes/genetic alterations targeted by the panel). For molecular categories defined in WHO [5], detection rates were estimated to be ~70% and ~92%, respectively. While combining these methods increased the diagnostic yield to approximately 98% for WHO-defined molecular categories (~93% for all known and potential AML-defining genetic drivers), it requires two separate sample collections, in contrast to a single collection for iWGS-WTS (Supplementary Fig. S15).

#### Additional pathogenic/likely pathogenic genetic aberrations co-occurring with driver genetic alterations

**Fig. 4:**
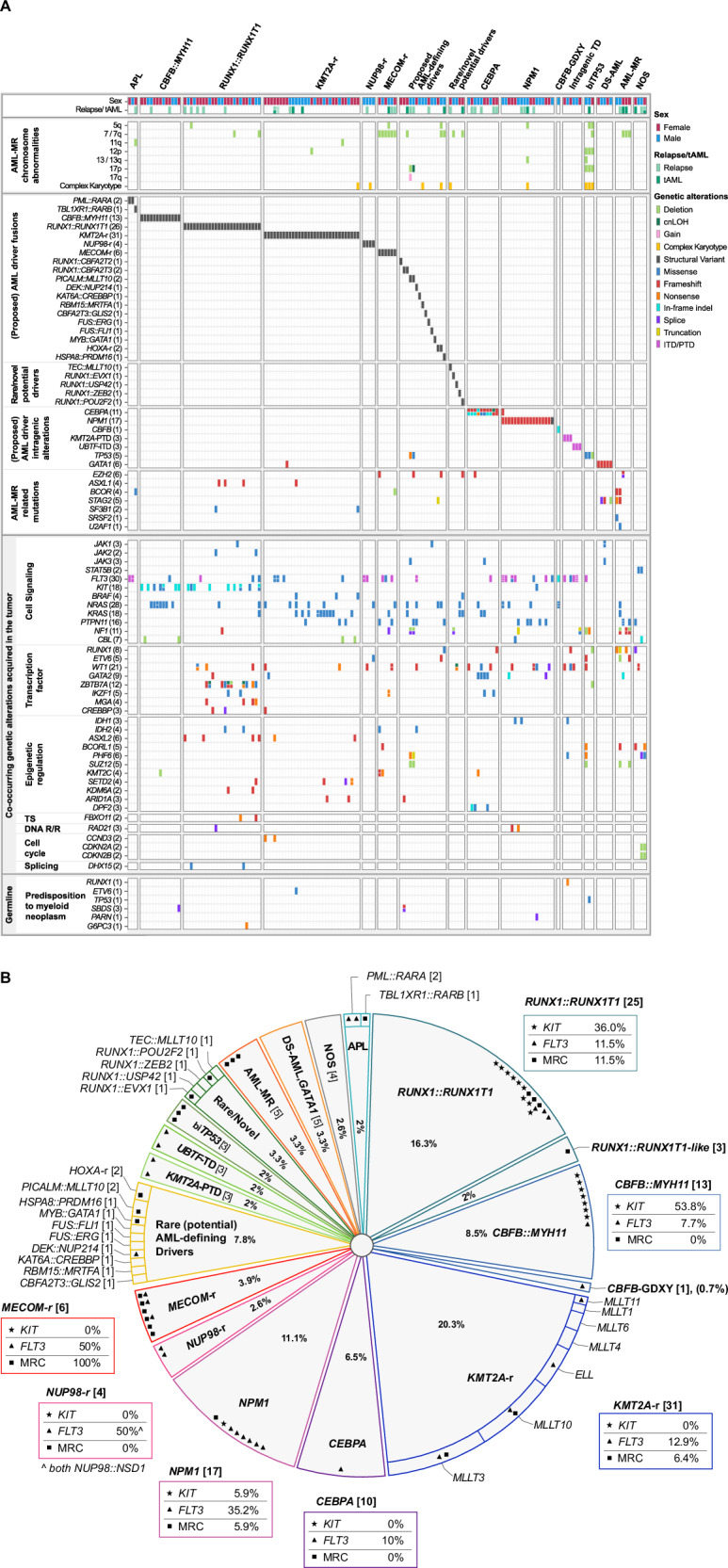
Genomic landscape of cases in the study cohort. **A** Oncoprint summarizing pathogenic/likely pathogenic (P/LP) tumor-acquired genomic alterations and selected germline variants identified in 153 AML cases using the integrated WGS and WTS (iWGS-WTS) approach. Patients’ sex, AML post cytotoxic therapy (a.k.a. therapy-related AML, tAML), and relapsed cases are indicated at the top, while germline-origin P/LP variants in genes associated with established genetic tumor syndromes predisposing to myeloid neoplasms defined in WHO^5th^ are presented at the bottom. **Note:** 1) For the case (SJ030708) with germline-origin *TP53* pathogenic variant, the same variant was detected in the tumor sample at a much higher variant allele frequency due to an acquired 17p deletion. This variant is shown in both the tumor-acquired and germline sections; 2) for tumor-acquired co-occurring genetic alterations, only alterations affecting the same gene and observed in at least two cases within this study cohort are diplayed in the Oncoprint. **B** The pie chart breaks down all 153 AML cases into subtypes according to genetic drivers/potential drivers determined by iWGS-WTS. Selected additional genetic alterations with established relevance to risk assessment and/or therapeutic targeting are marked with distinct symbols within the slices. If a patient has multiple hits in the same gene, only one symbol is displayed. Their percentages, calculated relative to each respective subtype, are displayed in boxes adjacent to their corresponding subtype slice. Notably, iWGS-WTS identified activating alterations in *KIT* in 16 of 38 core-binding factor AML cases in this study. Although only exon 17 mutations in *KIT* have been established as a poor prognostic factor in *RUNX1::RUNX1T1*-AML [46], other *KIT* activating mutations may also serve as potential molecular targets for AML treatment, particularly in relapsed cases [47]. **Abbreviations**: AML-MR acute myeloid leukemia, myelodysplasia-related, APL acute promyelocytic leukemia, biTP53 biallelic *TP53* alterations, *CBFB*-GDXY recurrent in-frame insertion mutations in *CBFB*, leading to a GDXY amino acid sequence change at position D87, cnLOH copy neutral loss of heterozygosity, DNA R/R DNA replication and repair, DS-AML Down Syndrome-associated AML, ITD intragenic tandem duplication, i(17q) isochromosome 17q, PTD partial tandem duplication, MRC AML myelodysplasia-related cytogenetic alteration, NOS not otherwise specified (no known/proposed or predicted AML-defining genetic alteration detected), -r rearrangement, TD tandem duplication, TS tumor suppressor, tAML therapy-related AML (AML post cytotoxic therapy), WTS whole transcriptome sequencing.

Along with the detection of major genetic driver alterations, comprehensive genomic profiling uncovered additional genetic abnormalities (Fig. 4A), providing a detailed picture for drawing conclusions on prognosis and risk stratification. Among all 153 patients, 414 additional P/LP alterations (SNVs/indels, focal CNVs, *FLT3*-ITDs, non-driver SVs, adverse large chromosomal abnormalities) were detected in approximately 92% patients. Approximately 30% of these additional findings were of direct clinical relevance, influencing risk assessment and/or informing therapy decisions. For instance, patient SJ032210, an *NPM1*-AML case, exhibited deletions in 5q and 13q, both associated with poorer prognosis [33, 34]. Selected cooperating genetic alterations with established clinical relevance were summarized in Fig. 4B.

## Discussion

Despite the advantages of using WGS and WTS in cancer molecular diagnostics [35–40], their clinical adoption remains limited, largely due to technical concerns to ensure accurate and timely leukemia diagnostics. In our institution, combined analysis of WGS, WES, and WTS has been routinely used for the initial diagnosis of leukemia patients since proof of concept [40] and clinical validation were completed. This study represents the first systematic review of our clinical experience using WGS and WTS, focusing on pediatric AML and highlighting insights gained since its implementation. Importantly, all data was obtained during real-time diagnostic evaluations, minimizing recall bias and providing actionable insights for laboratories seeking to adopt this workflow. Through extensive comparisons between WGS, WES, WTS, targeted NGS panel, conventional molecular and cytogenetic tests, we demonstrated that iWGS-WTS is a robust diagnostic test for effectively identifying key genetic drivers and cooperating genetic lesions, enabling precise disease classification and risk assessment in pediatric AML.

While WGS offers an unbiased view of genomic abnormalities, WTS adds complementary information such as SNVs/indels in RNA transcripts, fusion transcript detection, ectopic oncogene expression, allele-specific expression, and global gene expression signatures, further enhancing decision-making. Overall, iWGS-WTS identified 99% of variants with VAF >8% and ~96% with VAF ≥5%, despite an average of 61.5x WGS coverage (~20-fold lower than targeted panel sequencing) with no significant difference observed between samples above or below 80x coverage threshold recommended in a previous study [41]. This improvement may be attributed to workflow improvements by integrating WTS data. However, WTS relies on the presence of genomic alterations in the transcriptome, making it less effective for events like enhancer hijacking, emphasizing the need for an integrated iWGS-WTS approach.

Conventional cytogenetics, crucial for detecting large chromosomal abnormalities, often misses cryptic and complex rearrangements. NGS outperformed conventional cytogenetics by resolving complex genomes, which often make karyotyping slow, subjective, and error-prone. In this study, 18 AML-driver gene fusions identified by iWGS-WTS were missed by karyotyping due to their cryptic or complex nature. Optical genome mapping (OGM) is another emerging tool, addressing some cytogenetic limitations by detecting genome-wide structural variants with a resolution of 500 bp–5 kb and CNVs at a resolution of ~500 kb [42]. However, OGM has notable limitations in detecting ploidy changes [42, 43] and cannot detect small-scale variants such as SNVs and indels. In contrast, WGS provides a “one-stop shop” for molecular diagnostics and benefits from integration with WTS to enhance accuracy.

WGS also enables high-resolution mapping of SV breakpoints associated with CNVs, enhancing confidence and sensitivity in detecting segmental CNVs [44]. In this study, combining CNV analysis with soft-clipped reads identified 12 small CNVs (≤10 kb) in AML-related genes, which SNP-array assays could miss due to resolution limitations [45]. However, WGS may struggle with subclonal numerical abnormalities (CNVs not associated with SVs) affecting <30–40% of cells. Unlike conventional cytogenetics, which provides single-cell resolution but is limited by metaphase cell selection, WGS offers a population-level overview. While WGS may miss clinically relevant numerical aberrations present in small cell population, none of the undetected subclonal events in this study were of established clinical significance.

**Fig. 5:**
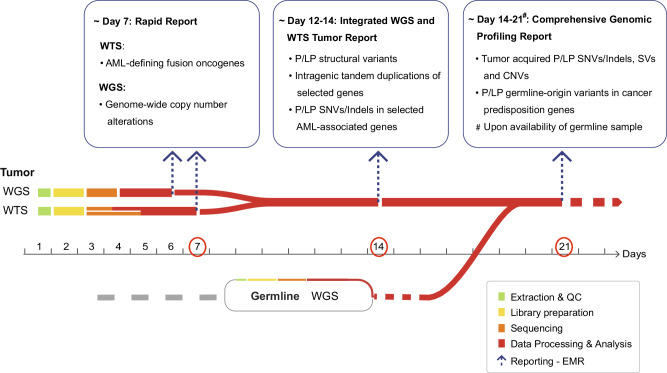
Advanced clinical workflow for the integrated WGS and WTS testing. A phased data processing and reporting approach for WGS, WTS and iWGS-WTS is shown along with timeline indicated. Segments in different colors along the timeline indicate varying steps of the process from sample acquisition/processing to data processing and analysis. Reports can be released in three phases, as indicated above: rapid tumor report by day 7, integrated iWGS-WTS tumor report focusing on selected alterations by day 14, and a comprehensive paired tumor-normal genomic profiling report by day 21. **Abbreviations**: CNV copy number variant, EMR electronic medical record, P/LP pathogenic/likely pathogenic, iWGS-WTS integrated WGS and WTS, QC quality control, SNV/Indel single nucleotide variant and small insertion-deletion, SNV single nucleotide variant, SV structural variant, WGS whole genome sequencing, WTS whole transcriptome sequencing.

Turnaround time (TAT) of tumor molecular profiling is critical for AML clinical decisions. Both rapid tumor diagnosis and comprehensive tumor/germline profiling are essential for accurate diagnosis and patient management [24, 38]. Throughout the duration of this study, we developed a phased approach to enable early reporting of key tumor-related genetic alterations while conducting paired tumor-normal analysis for a full somatic and germline report. The workflow (Fig. 5), outlining both current practices and components under implementation, allows reporting of most AML-defining drivers and LS-CNVs within seven days, rare or novel fusions and cooperating variants by day 14, and a complete tumor and germline profile within 2–3 weeks upon availability of a matched germline sample.

Unlike the standard AML diagnostic workup (CC + AML-FISH and NGS panels), which requires multiple sample collections and tests, iWGS-WTS streamlines the process, reducing complexity and potential cost. WGS and WTS also enable rapid adoption of new diagnostic markers through bioinformatics updates without extensive assay development. Previously generated data can be reanalyzed for emerging biomarkers, crucial in molecular oncology, where samples are often limited.

Our successful implementation of iWGS-WTS in pediatric AML diagnosis demonstrates its potential to streamline resource use, accelerate disease classification and treatment planning, and leverage genomic data for future research and global data sharing. This study provides strong evidence for replacing standard diagnostic approaches with iWGS-WTS for pediatric AML and highlights its promise for broader application in pediatric cancer diagnostics.

## Supplementary information

Supplementary Information

Dataset_Supplementary Tables

## Data Availability

The next generation sequencing data for the current study are available under the accession number SJC-PB-1035, at the St. Jude Cloud Genomics Platform (https://platform.stjude.cloud/data/publications?publication_accession=SJC-PB-1035). Key to identifiers can be found in Supplementary Table S10. The work was funded by the American Lebanese and Syrian Associated Charities of St. Jude Children’s Research Hospital and R01 CA276079 (JMK). JMK holds a Career Award for Medical Scientists from the Burroughs Welcome Fund. KEN is supported by R01 R01CA24145. The content, however, does not necessarily represent the official views of the NIH and is solely the responsibility of the authors. We thank staff at SJCRH Biorepository and the Cytogenetics Shared Resource, which is supported through the St. Jude Comprehensive Cancer Center (P30-CA21765).
