# Supplementary material for: Clinical experience of using integrated whole genome and transcriptome sequencing as a framework for pediatric and adolescent acute myeloid leukemia diagnosis and risk assessment
Source: Leukemia. 2025 Oct 7;39(12):2946–56. doi: 10.1038/s41375-025-02774-5 (PMC12634442; doi:10.1038/s41375-025-02774-5)
Supplement: Supplementary file 1 — Supplementary Information [file 41375_2025_2774_MOESM1_ESM.pdf]

Supplementary Information

Clinical Experience of Using Integrated Whole Genome and Transcriptome Sequencing as a Framework for Pediatric and Adolescent AML Diagnosis and Risk Assessment

Voss R., Pastor Loyola V. *et al.*

Section 1. Supplementary Material and Methods

1.1 Cell line information

1.2 WGS, WES, and WTS library preparation and sequencing

1.3 Sequencing data analysis, variant calling and classification

1.4 Targeted DNA sequencing

1.5 Conventional cytogenetic analysis

Section 2. Supplementary Figures

2.1 Figure S1. Variant process and auto calling rules

2.2 Figure S2. Whole genome sequencing (WGS) QC metrics

2.3 Figure S3. Whole transcriptome sequencing (WTS) QC metrics

2.4 Figure S4. Representative cases illustrating focal copy number variant (CNV) detection by whole genome sequencing (WGS)

2.5 Figure S5. *KMT2A::ELL* fusions in cases SJ031554 and SJ031359

2.6 Figure S6. Cases with enhancer hijacking structural alterations

2.7 Figure S7. Two cases (SJ030153, SJ030431) with t(5;14)(q35;q32.2) by WGS

2.8 Figure S8. Case SJ031206 with *HSPA8::PRDM16* fusion

2.9 Figure S9. Global gene expression profiling highlighting bi*CEBPA*/smbZIP-*CEBPA* AML molecular subtype

2.10 Figure S10. Global gene expression profiling of cases with *RUNX1*-rearrangements

2.11 Figure S11. Example cases demonstrating complex genomes resolved by integrated WGS and WTS (iWGS-WTS)

2.12 Figure S12. Overview of the comparison of whole genome sequencing (WGS) and conventional cytogenetics in the detection of large-scale copy number variants (CNVs)

2.13 Figure S13. Abnormal chromosomes observed by conventional karyotype analysis further clarified by whole genome sequencing (WGS)

2.14 Figure S14. Detection sensitivity of large scale copy number alterations in dilution series

2.15 Figure S15. Comparison of iWGS-WTS, cytogenetics and NGS panel testing in the molecular diagnosis and classification of AML

Section 3. Supplementary References

Section 1. Supplementary Material and Methods

1.1 Cell line information

A publicly available immortal cancer cell line COLO-829 (CRL-1974, ATCC, USA) and COLO-829BL (CRL-1980, ATCC, USA), a lymphoblastoid cell line derived from the same patient, were obtained from ATCC. The WGS performance for detecting large-scale copy number variants (LS-CNVs) was also evaluated through a series of dilution experiments using COLO829 tumor cell line and its matched normal COLO829L at tumor DNA purities of 100%, 40% and 30%, respectively. Large-scale (>5 Mb) copy number variants of 1 or 2 copy gains of chromosomes 1q, 2, 3, 7, 12 and 19 were chosen for evaluation (1).

1.2 WGS, WES, and WTS library preparation and sequencing

DNA was directly extracted from fresh samples or mononuclear cells that were purified from bone marrow or peripheral blood by density gradient centrifugation (Ficoll) using phenol chloroform or Chemagic 360 auto-extraction (Revvity chemagen Technologie GmbH, USA). RNA was extracted using TRIzol reagent (Thermo Fisher Scientific, USA) or by Chemagic 360 auto-extraction. DNA and RNA integrity was assessed by agarose gel electrophoresis and Agilent 2100 BioAnalyzer (Agilent Technologies, USA), respectively. DNA and RNA concentrations were measured using a Qubit Fluorometer (Thermo Fisher Scientific, USA). The WGS, WES, and WTS tests were performed as previously described (2). In brief, high molecular weight DNA was extracted, and 1μg and 150ng DNA from each sample was used for WGS and WES, respectively. The minimum total RNA input for WTS library preparation was 250ng. Sequencing libraries were constructed using the TruSeq DNA PCR-Free sample preparation kit (Illumina, Inc.,USA) for WGS, the TruSeq exome enrichment kit v1 (Illumina, Inc., USA) for WES, and the TruSeq Stranded Total RNA Kit (Illumina, Inc., USA) for WTS. All sequencing was done with HiSeq4000 (2016 – early 2020) or NovaSeq 6000 (2020 and onward).

1.3 Sequencing data analysis, variant calling and classification

Sequencing data were processed by our computational bioinformatics pipeline as previously described (2). The variant output from this pipeline was further processed following our auto-calling rules (Supplementary Figure S1) and valid alterations were carefully reviewed by molecular diagnostic professionals in the context of each complete case for variant classification. Germline variants were classified according to the ACMG/AMP guidelines (3), with additional input from ClinGen Variant Expert Panels (VEPs) where available (4). In parallel with the ACMG framework, we applied the point-based classification system (5) as supportive evidence to help

resolve classification in cases with conflicting or limited data. Germline-origin pathogenic and likely pathogenic variants as well as variants of unknown clinical significance were included in clinical reports.

For somatic variants, evidence-based categorization was primarily performed in accordance with guidelines from the Association for Molecular Pathology (AMP), American Society of Clinical Oncology (ASCO), and College of American Pathologists (CAP) (6). Additionally, public databases and internal data/experience were considered when necessary (7,8). Only tier I variants (with strong clinical significance) and tier II variants (with potential clinical significance) were included in clinical reports. Tier III variants, classified as having unknown clinical significance, were excluded due to the large volume of data generated by WGS and WTS tumor genomic and transcriptomic analyses. In this study, tier I and tier II variants were described as pathogenic or likely pathogenic (P/LP).

Gene expression analysis was performed to evaluate supporting evidence for detected sequence variants and to assist in molecular classification. To visualize the expression of any gene of interest across samples, normalized expression values were generated from raw sequencing data. Read-count data for each case was generated using HTSeq version 0.11.2 (9), with gene annotation based on GENCODE v31. Gene expression analysis was performed using the DEseq2 (10) and sva (11) R packages. To normalize raw HTSeq counts, variance stabilizing transformation (VST) was applied to the data, followed by batch correction using the ComBAT function from the sva package (11). Expression values were transformed separately for each gene to account for the mean–variance relationship across samples. As a result, baseline values corresponding to zero raw counts vary slightly between genes. Global gene expression profiling was conducted using UMAP (Uniform Manifold Approximation and Projection) analysis as previously described (12). Clustering is shown in the context of an extended cohort of 419 AML patients including 153 AML patients of this study cohort (indicated as diamonds) and 266 cases of a reference cohort generated from data available through St. Jude Cloud (https://www.stjude.cloud).

1.4 Targeted DNA sequencing

Thirty-nine patients were tested by Archer™ VARIANTPlex™ Myeloid NGS panel (Integrated DNA Technologies, USA). The sequencing library was prepared with 50-100ng DNA input and sequenced on MiSeqDx (Illumina, USA). Sequencing data were analyzed by Archer® Analysis software (version 6.2) and variant calling was determined based on the default criteria provided

by the vendor. This targeted NGS panel covers 75 genes and can detect SNVs and small indels at a variant allele frequency (VAF) as low as 3%.

1.5 Conventional cytogenetic analysis

Conventional cytogenetic analysis was performed on 24-hour unstimulated bone marrow cultures with or without synchronization according to standard procedures. At least 20 metaphases were analyzed, and karyotypes were interpreted according to the International System for Human Cytogenetic Nomenclature. Fluorescence in-situ hybridization (FISH) was performed as needed.

Section 2. Supplementary Figures

Pages 7-38

Figure S1. Variant process and auto calling rules

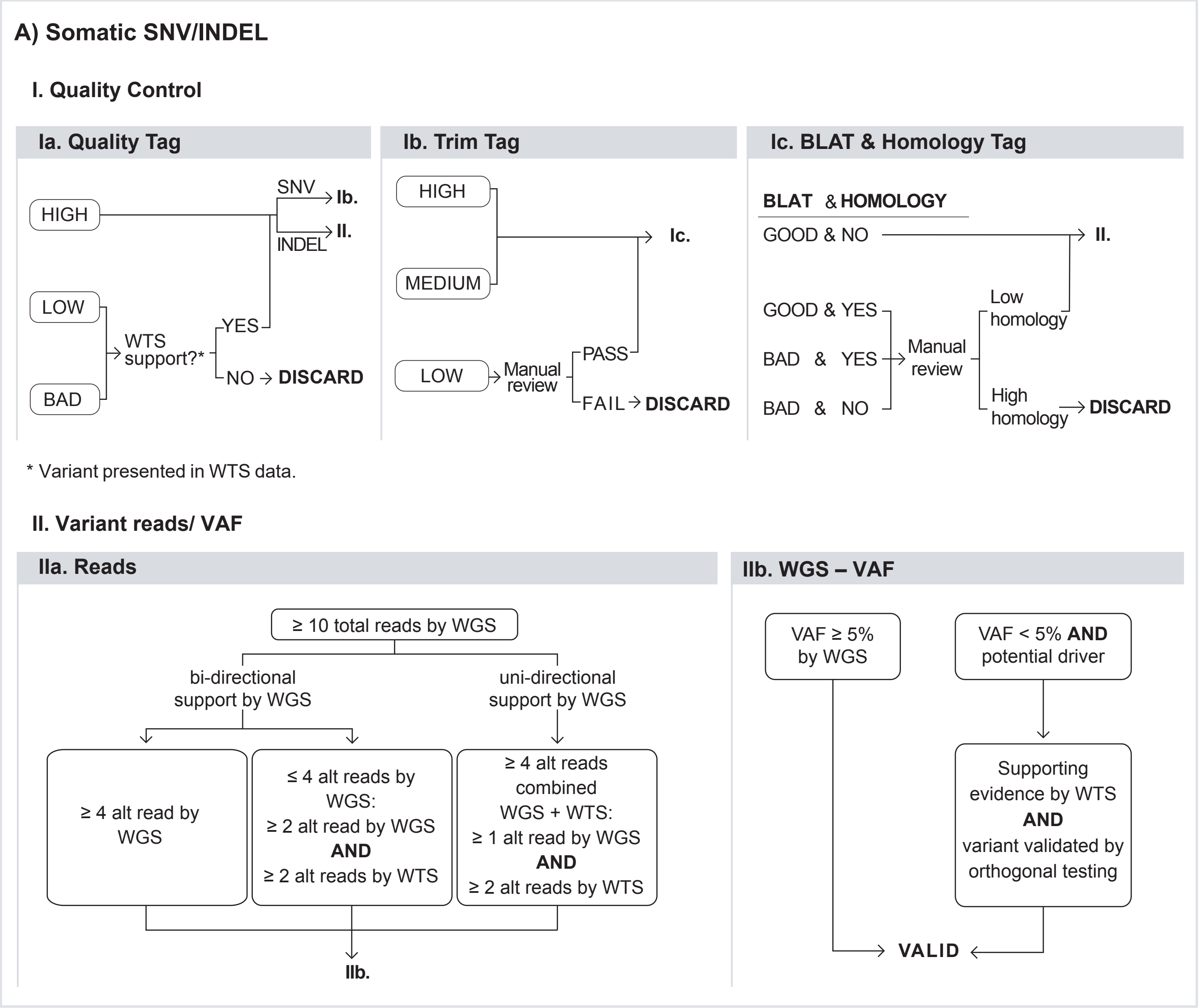

Figure S1. A) SNVs/Indels in tumor testing. This figure details the process and curation rules for Single Nucleotide Variants (SNVs) and small insertion deletions (indels) for integrated Whole Genome and Whole Transcriptome Sequencing (iWGS-WTS). The process starts with rigorous quality control **(I)** involving three specific tags: (Ia) Quality Tag to ensure base quality, (Ib) TrimTag to ensure proper trimming of sequencing reads, and (Ic) BLAT Tag to confirm correct genomic alignment; Ib and Ic only apply for SNVs. After quality control, variant verification is based on mutation reads **(II)**. IIa) A minimum of 10 total reads and at least four alternative (alt) reads are required, which may be substituted by alt reads in WTS data. If WGS alt reads show only one-sided support, at least two supporting alternative reads by WTS are required. IIb) Variants with a Variant Allele Frequency (VAF) of ≥ 5% are considered reliable and classified, and SNVs with a VAF below 5% require verification by an alternative sequencing method (targeted NGS or WES) if WTS evidence is present (IIc). Abbreviations: BLAT, BLAST-like alignment tool.

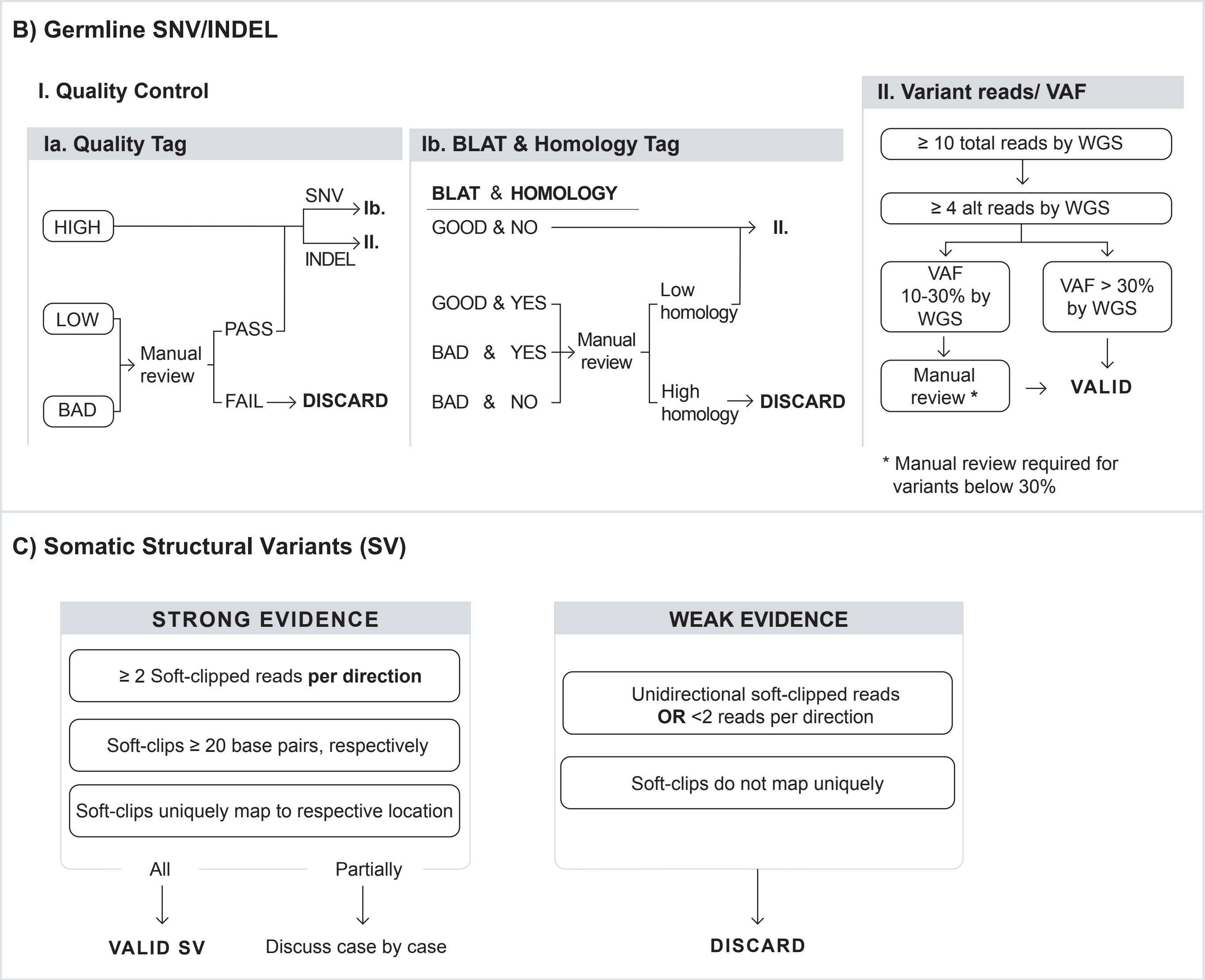

Figure S1. B) Germline SNV/INDEL. For germline variants, the workflow begins with quality control (I), similar to that used for tumor testing but includes an additional ‘Homology Tag’ to account for sequence homology, reducing false positives from repetitive regions. After passing quality control, germline variants are evaluated based on the total number of alternative (alt) and reference reads. II) A minimum of 10 total reads and more than four alternative reads at a variant allele frequency (VAF) of at least 30% are required in WGS data before proceeding to variant classification. Variants with a VAF between 10-30% require manual review. **C) Somatic structural variants.** This figure outlines the curation for Structural Variants (SV) in tumor Whole Genome Sequencing (WGS). For strong evidence of a valid SV, at least two soft-clipped reads must be present on both sides of the fusion junction, with each soft-clip being at least 20 base pairs long and uniquely mapping to the genomic location. If only one-sided support or fewer than two reads are present, the variant is classified as weak evidence, requiring further manual review. Whole transcriptome sequencing (WTS) may provide additional evidence. Variants that fail to meet any diagnostic criteria are discarded, while borderline cases undergo case-by-case discussion for curation. BLAT, BLAST-like alignment tool.

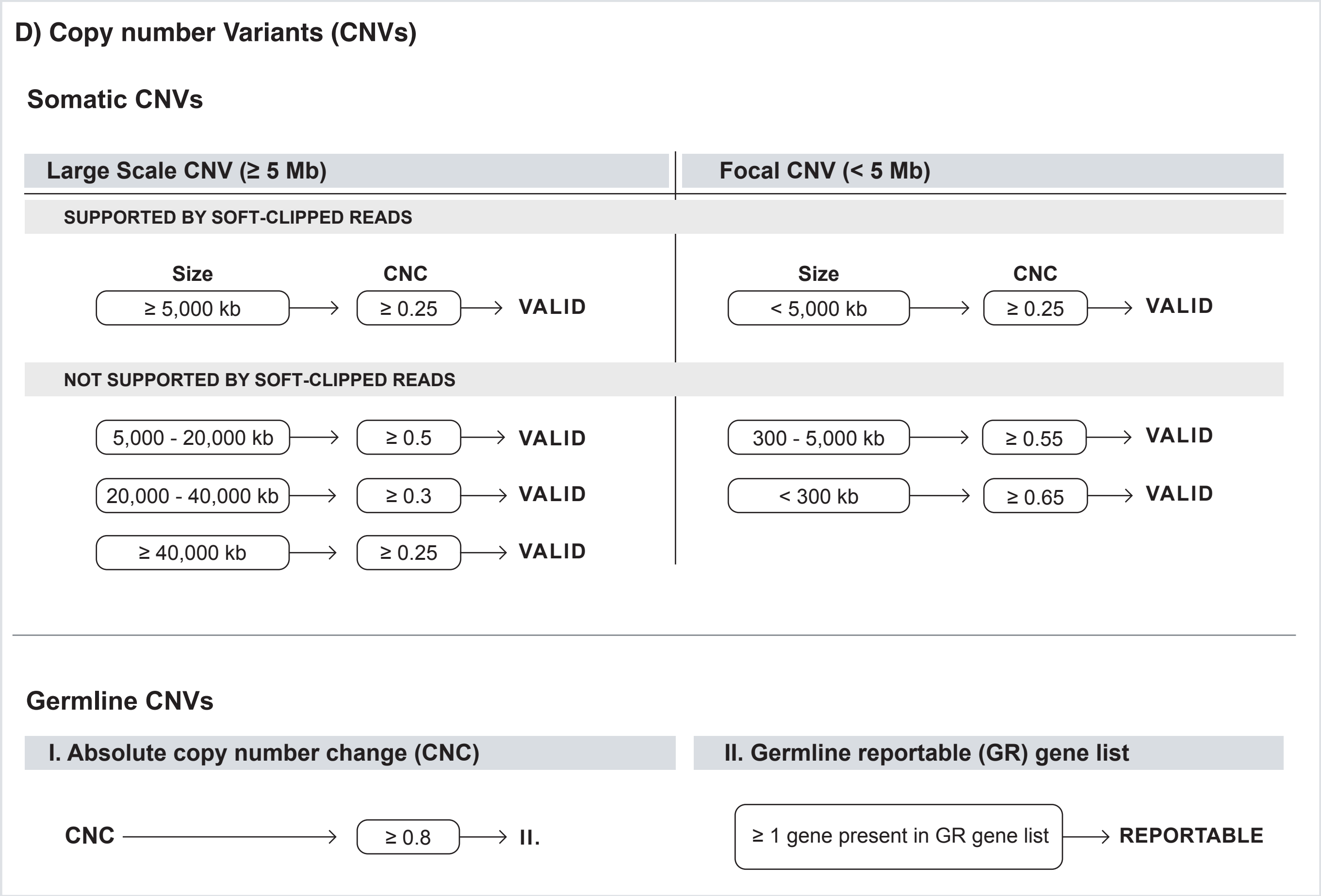

Figure S1. D) Copy number Variants (CNV) detection by whole genome sequencing (WGS). Upper panel: Somatic CNVs. Curation rules for CNVs detected in tumor Whole Genome Sequencing (WGS): Large-scale CNVs, defined as larger than 5,000 kb, require copy number change (CNC) thresholds of ≥ 0.25 to 0.5 depending on their size and whether they are associated with a structural variant (SV). Focal CNVs (smaller than 5,000 kb) require stricter thresholds for CNVs not associated with SVs. Soft-clipped reads of an associated SV provide additional evidence, particularly for segmental CNVs. **Lower panel: Germline CNVs.** The focus of germline CNV analysis lies in the detection of CNVs, which cover genes associated with cancer or bone marrow failure (BMF) predisposition. First, CNC is evaluated, and if cut-off is met, Germline Reportable (GR) gene list (Supplementary Table S4A) is consulted to determine whether a GR gene lies within the copy number alteration. If so, the event may be classified per ACMG recommended rules.

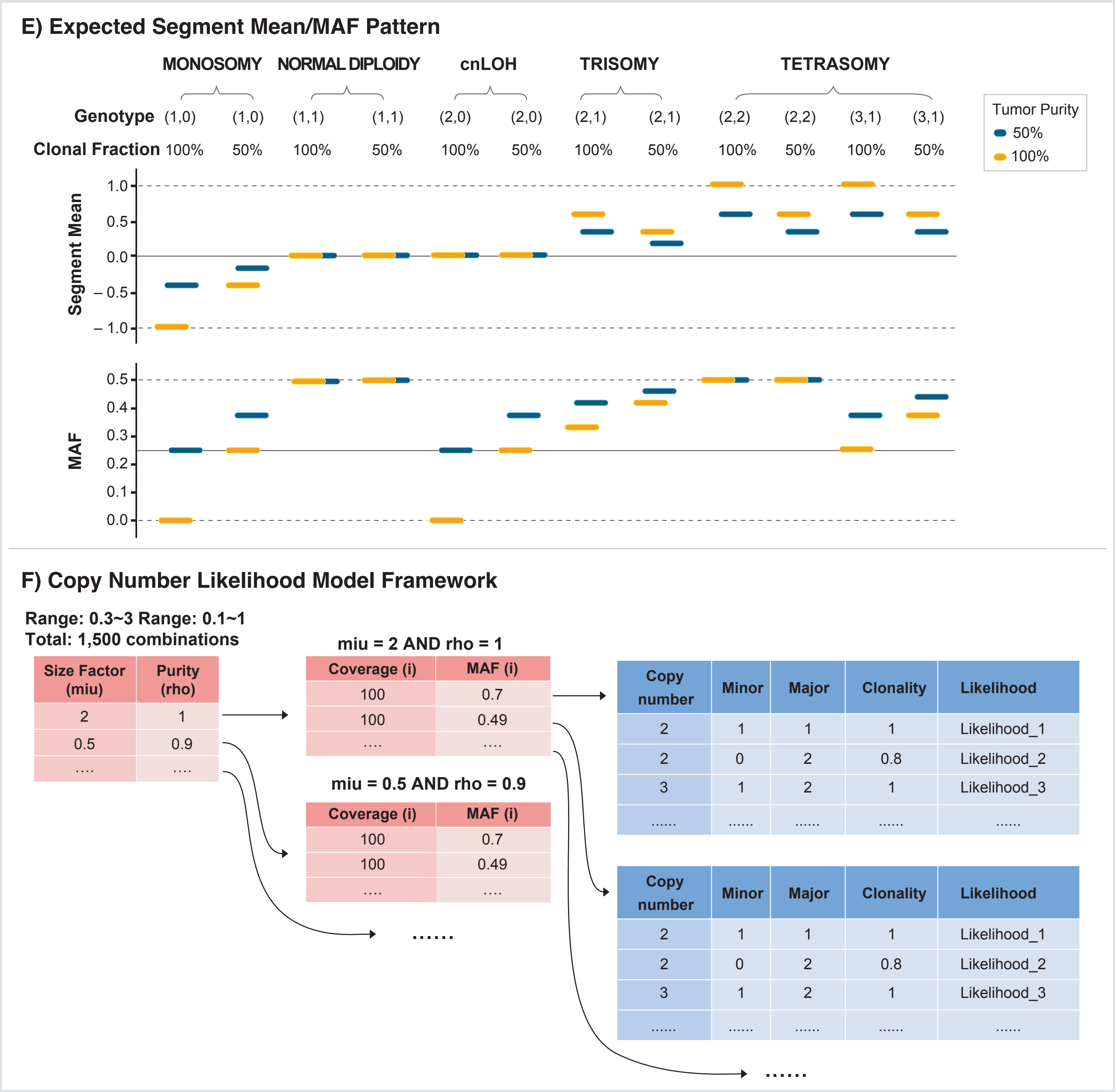

Figure S1. E) and F) Estimate copy number status based on sequencing coverage and minor allele frequency (MAF). Coverage and allelic imbalance information derived from WGS were integrated to assess copy number status and numerical chromosome abnormalities, using an approach based on established methodologies (PMID: 22544022). A schematic reference was generated to aid in the interpretation of allelic copy number status across varying tumor purity and clonal fractions (E). In normal diploid regions, a 50:50 allelic distribution is typically expected for heterozygous SNPs. Deviations from this pattern indicate allel-specific imbalances, which, when integrated with coverage data, improve the accuracy and sensitivity of copy number estimation. **E) Illustrative reference** – Expected Segment Mean (log2 coverage ratio) and MAF are presented for each configuration of genotypes along with clonal fraction and tumor purity. For instance, a clonal (100% clonal fraction) monosomy (genotype: 1,0) at 100% tumor purity yields segment mean of -1 and MAF equals 0, while the same CNV in a sample of 50% tumor purity may show a reduced segment mean of -0.415 and MAF

at 0.25. Low clonal fraction further impacts segment mean and MAF. **F) Copy Number Likelihood Model.** A conceptual overview of our procedure of using the model to identify best-fit parameters to determine baseline diploidy and assess CNVs in relation to the baseline. Abbreviations: cnLOH, copy neutral loss of heterozygosity; CNV, copy number variant; MAF, minor allele frequency; SNP, single nucleotide polymorphism.

Figure S2. Whole genome sequencing (WGS) QC metrics

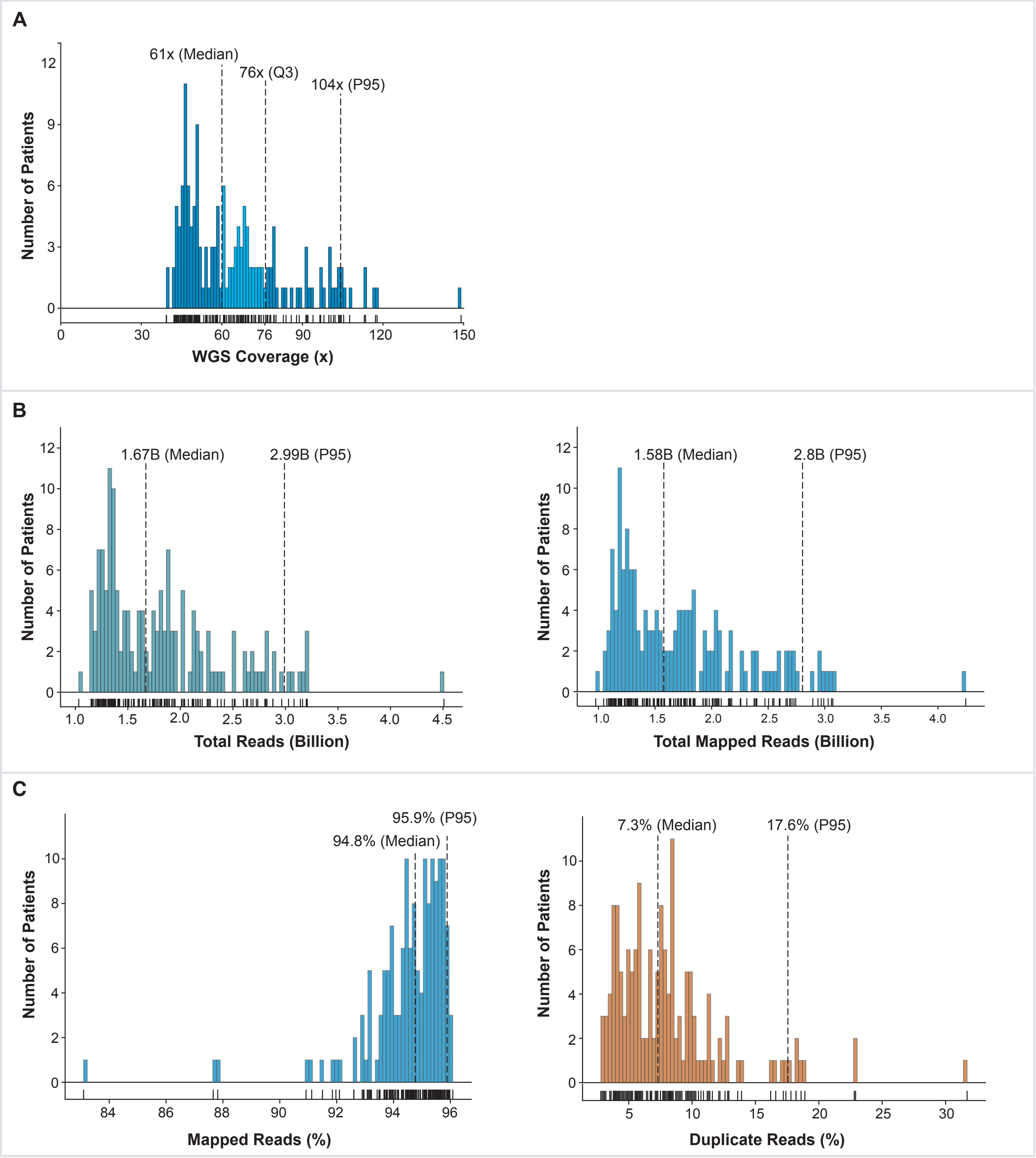

Figure S2. A) WGS average coverage across the study cohort. Median, third quartile (Q3) and 95th percentile (P95) are shown as black, dashed lines. **B)** Tallied total number of reads across patients is shown on left, total mapped reads on the right. Median and P95 are indicated by dashed lines. **C)** The percentage of mapped reads are shown on the left, the percentage of duplicate reads on the right.

Figure S3. Whole transcriptome sequencing (WTS) QC metrics

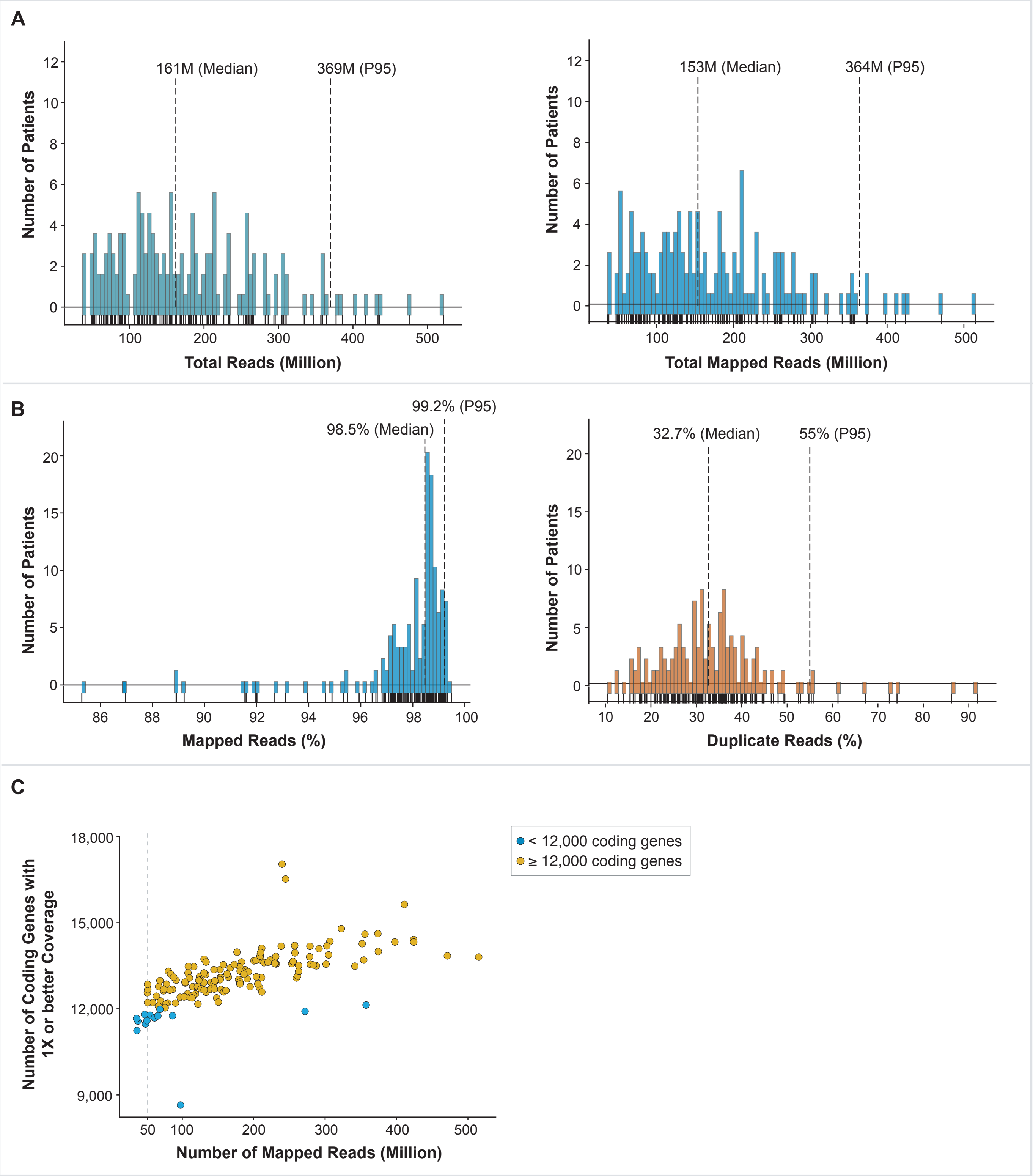

Figure S3. A) Tallied total number of whole transcriptome sequencing (WTS) reads across patients is shown on the left, total mapped reads on the right. Median values and 95th percentile (P95) are indicated by dashed lines. **B)** Percent of mapped reads are shown on the left, percent of duplicate reads on the right. Mapping and duplicate rate are indicated by median respectively. The 95th percentiles are shown by dashed lines. **C)** Number of mapped reads vs. number of coding genes with 1x or better coverage are depicted for all 153 patients. Gencode v19 was used as reference gene-list.

Figure S4. Representative cases illustrating focal copy number variant (CNV) detection by whole genome sequencing (WGS)

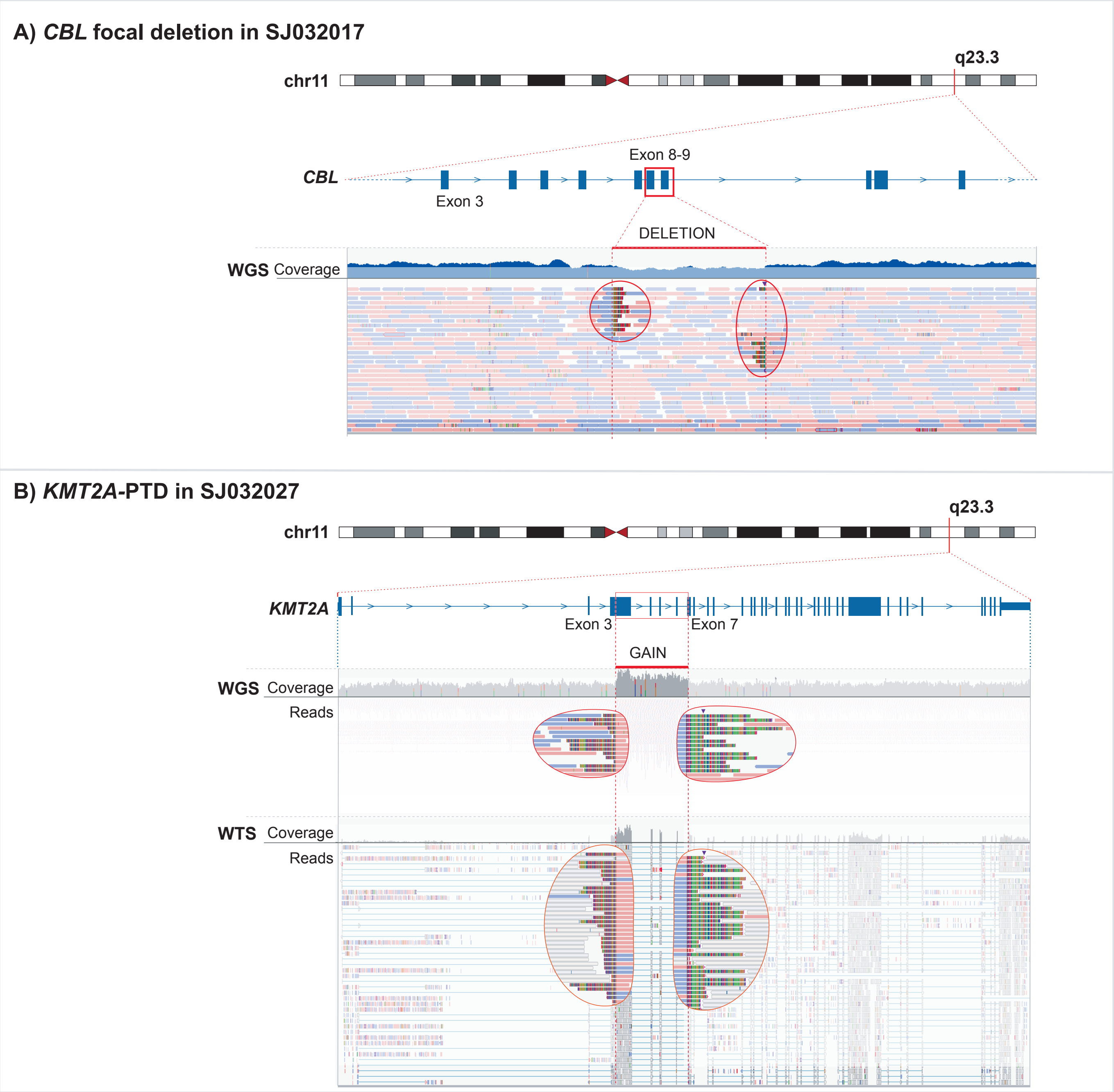

Figure S4. Intragenic CNVs were considered valid when supported by both WGS-derived read-depth changes and soft-clipped reads (see Supplementary Fig. S1D for diagnostic criteria). **A)** Intragenic deletion in *CBL* (NM_005188.3) in a representative patient, affecting exons 8 and 9. Soft-clipped bases demarcating the deletion are circled, a corresponding drop in WGS coverage is visible between these points (red line). **B) *KMT2A*-PDT.** Partial tandem duplication (PTD) affecting exons 3-7 of *KMT2A* (NM_005933.4) in a representative patient, SJ032027. Soft-clipped reads from both WGS and WTS are circled in the affected region. An increase in WGS coverage over the duplicated segment is evident on the coverage track (red line). Abbreviations: CNV, copy number variant; WGS, whole genome sequencing; WTS, whole transcriptome sequencing.

Figure S5. *KMT2A::ELL* fusions in cases SJ031554 and SJ031359

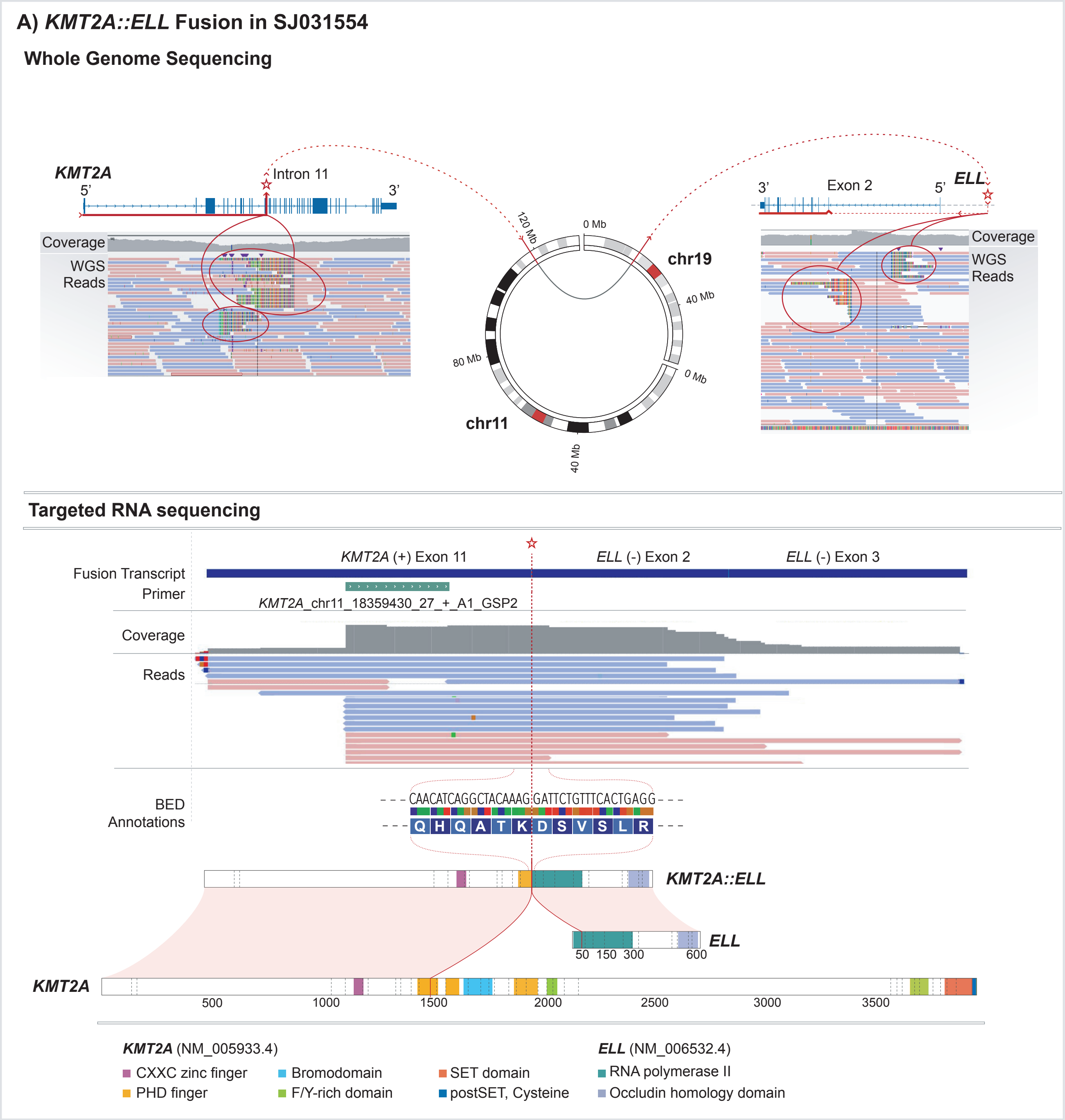

Figure S5. A) SJ031554 – the CIRCOS plot illustrates structural variant breakpoints from WGS on chromosomes 11 and 19. WGS soft-clipped reads indicate a fusion between *KMT2A* intron 11 and a region ~3.4 kb upstream of *ELL* at 19p13.3. Although WTS did not yield sufficient diagnostic evidence for a *KMT2A::ELL* fusion, manual review of the WTS BAM file revealed a single read supporting an in-frame fusion of *KMT2A* exon 11 to *ELL* exon 2. This fusion was subsequently confirmed by targeted RNA sequencing using the Archer™ FUSIONPlex™ Pan-Heme panel (IDT, USA). A partial sequence of the fusion transcript and a schematic of the predicted chimeric protein are shown, the fusion junction is indicated as a red star.

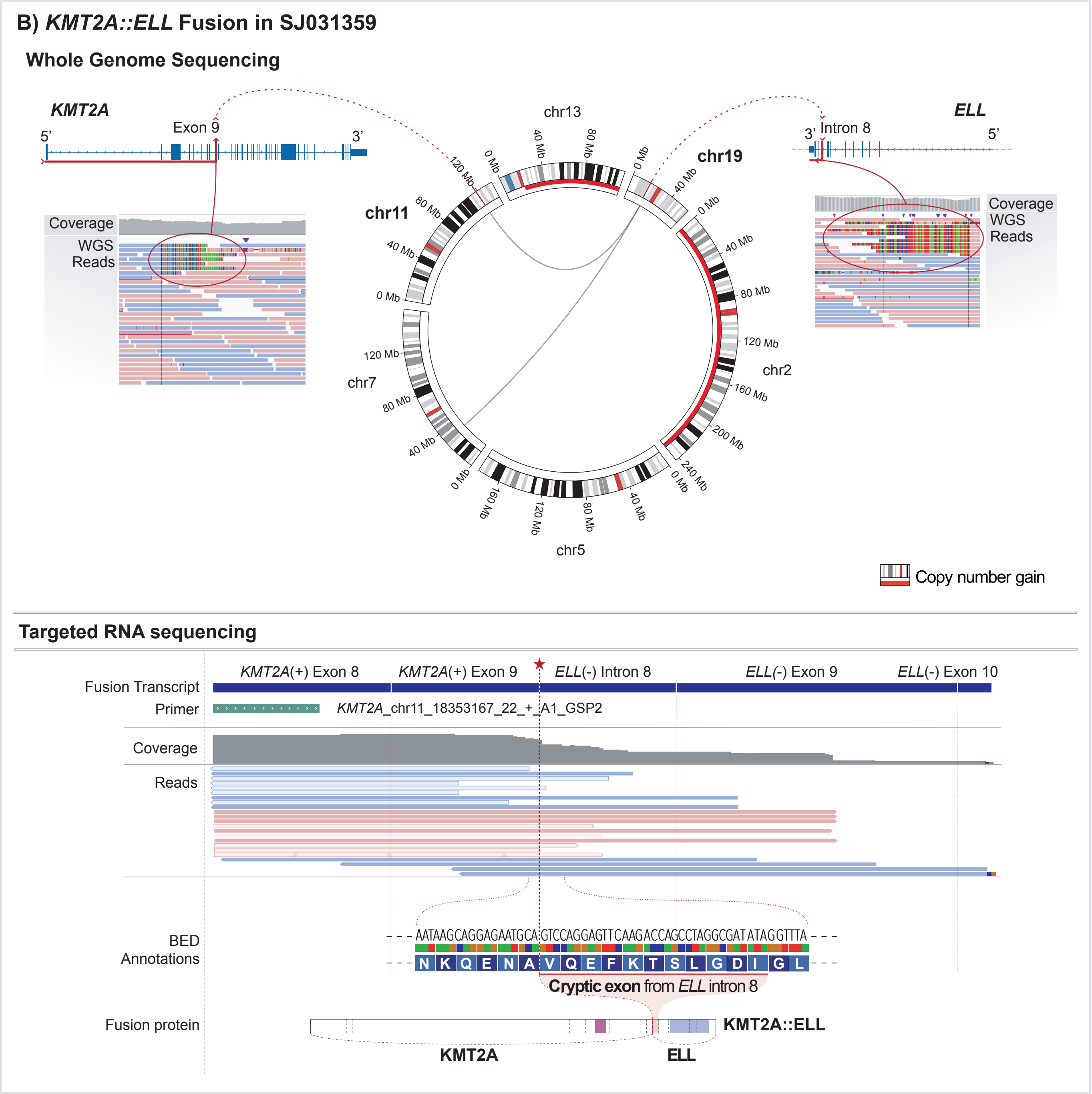

Figure S5. B) SJ031359. The CIRCOS plot shows structural variant breakpoints from WGS on chromosomes 11 and 19, indicating a *KMT2A::ELL* fusion. No supporting reads for a chimeric *KMT2A::ELL* transcript were observed by WTS. Targeted RNA sequencing revealed a rare in-frame fusion isoform, joining *KMT2A* exon 9 to a cryptic exon within *ELL* intron 8, followed by *ELL* exon 9. A partial sequence of the fusion transcript and a schematic diagram of the predicted chimeric protein are shown, the fusion junction is indicated by a red star. See Figure S5A for domain annotations of the predicted fusion protein. *KMT2A* transcript: NM_005933.4; *ELL* transcript: NM_006532.4. Abbreviations: WGS, whole genome sequencing; WTS, whole transcriptome sequencing.

Figure S6. Cases with enhancer hijacking structural alterations

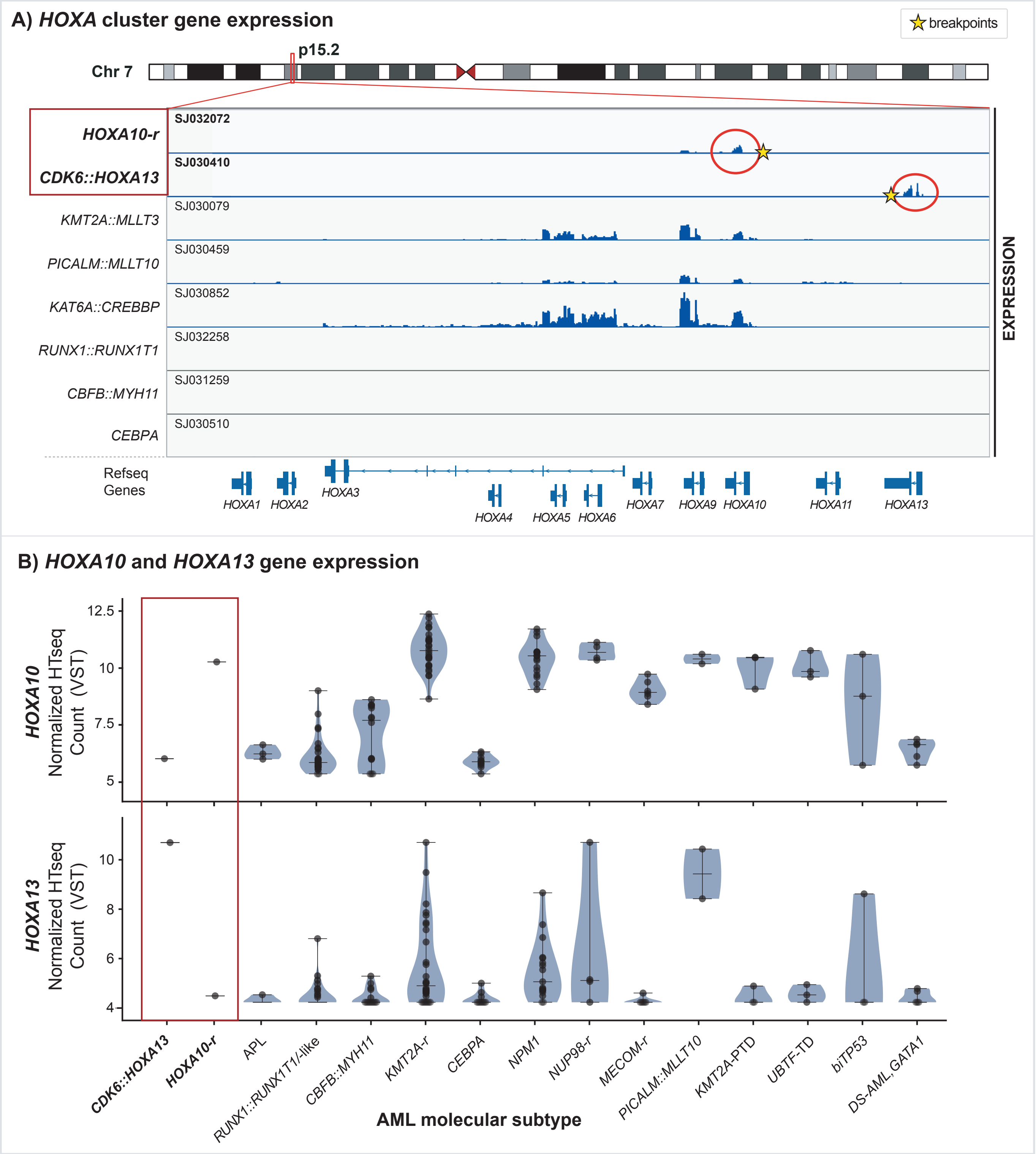

Figure S6. A) WTS coverage (expression) of *HOXA* gene cluster is shown for two patients with *HOXA*-r, in contrast to selected patients with other AML-driver genetic alterations: three patients from molecular subtypes of which high *HOXA gene* expression is expected (*KMT2A*-r, *PICALM::MLLT10, KAT6A::CREBBP*), as well as three patients from subtypes of which no increased expression of *HOXA* cluster genes is expected (*RUNX1::RUNX1T1, CBFB::MYH11,* bi*CEBPA/sm*bZIP-*CEBPA*). Stars indicate breakpoints. **B) Normalized expression of *HOXA10* and *HOXA13* genes.** *HOXA*-rearranged

AML cases are shown on the left side of the plot, additional AML-subtypes with two or more cases from this study cohort are shown for comparison. Expression values are shown as variance-stabilized counts (VST) derived from HTSeq counts, which are not comparable between genes due to gene-specific dispersion estimates and batch correction (see Supplementary Methods). In this dataset, a baseline of 4.0 (*HOXA10*) and 4.2 (*HOXA13*) corresponds to a raw HTSeq count of 0. Abbreviations: APL, acute promyelocytic leukemia; bi*TP53*, biallelic *TP53* alterations; *CEBPA*, bi*CEBPA*/smbZIP-*CEBPA*; chr, chromosome; DS-AML, Down Syndrome related AML; PTD, partial tandem duplication; TD, tandem duplication; WTS, whole transcriptome sequencing.

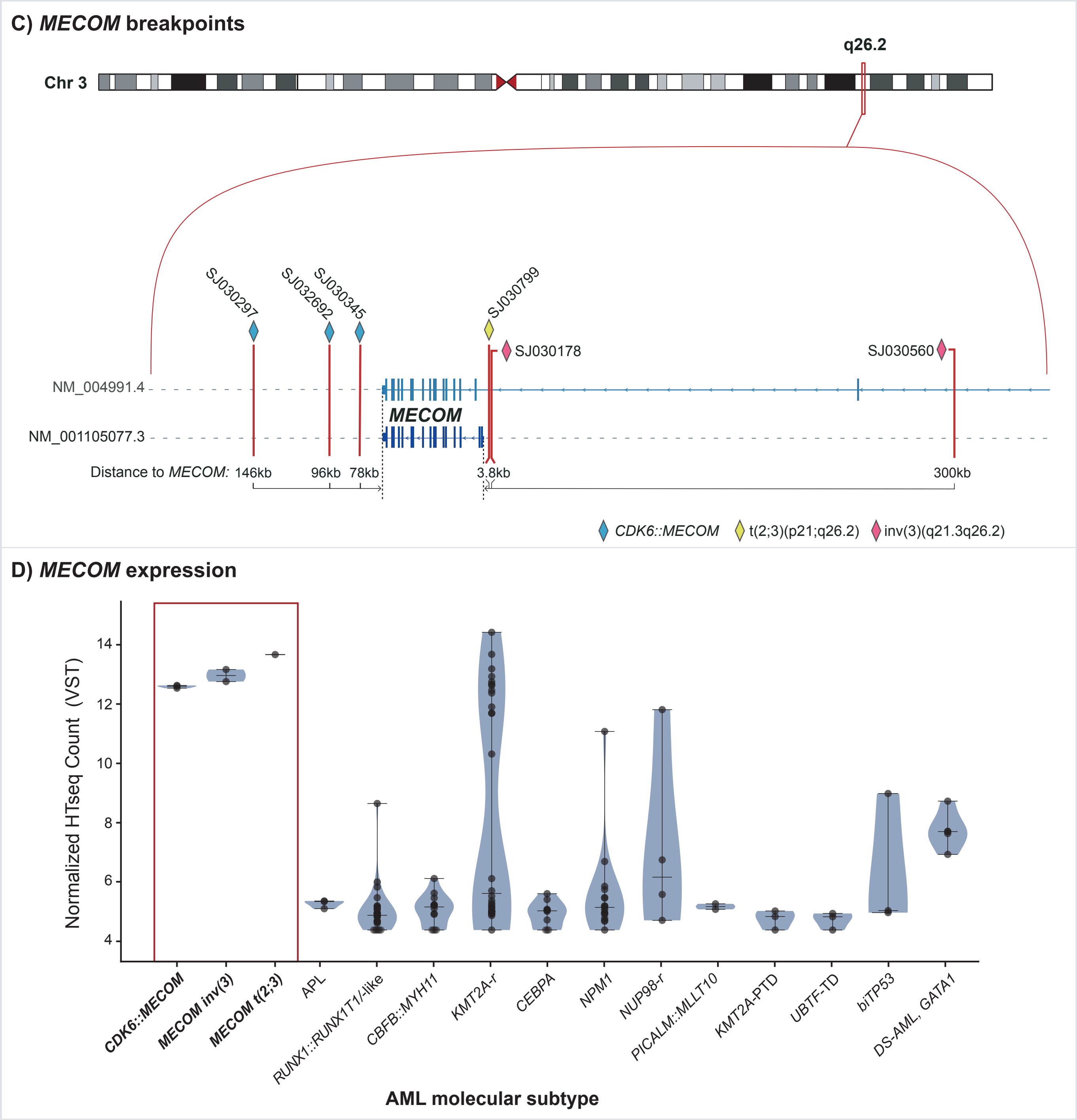

Figure S6. C) Breakpoints in proximity to *MECOM*. Affected region in chromosome 7 is highlighted and *MECOM* transcripts are shown schematically, with lines indicating breakpoints and colored diamonds representing subtypes of *MECOM* rearrangements for respective patients. Distance to *MECOM* 5' and 3' ends are shown below. **D) Normalized expression of *MECOM*.** *MECOM* -r cases are shown on the left, other AML subtypes from this cohort are shown as comparison. Expression values are shown as variance-stabilized counts (VST) derived from HTSeq counts. In this dataset, a baseline of 4.4 corresponds to a raw HTSeq count of 0. **Abbreviations:** APL, acute promyelocytic leukemia; bi*TP53*, biallelic *TP53* alterations; *CEBPA*, bi*CEBPA*/smbZIP-*CEBPA*; chr, chromosome; DS-AML, Down Syndrome related AML; kb, kilobases; PTD, partial tandem duplication; TD, tandem duplication;

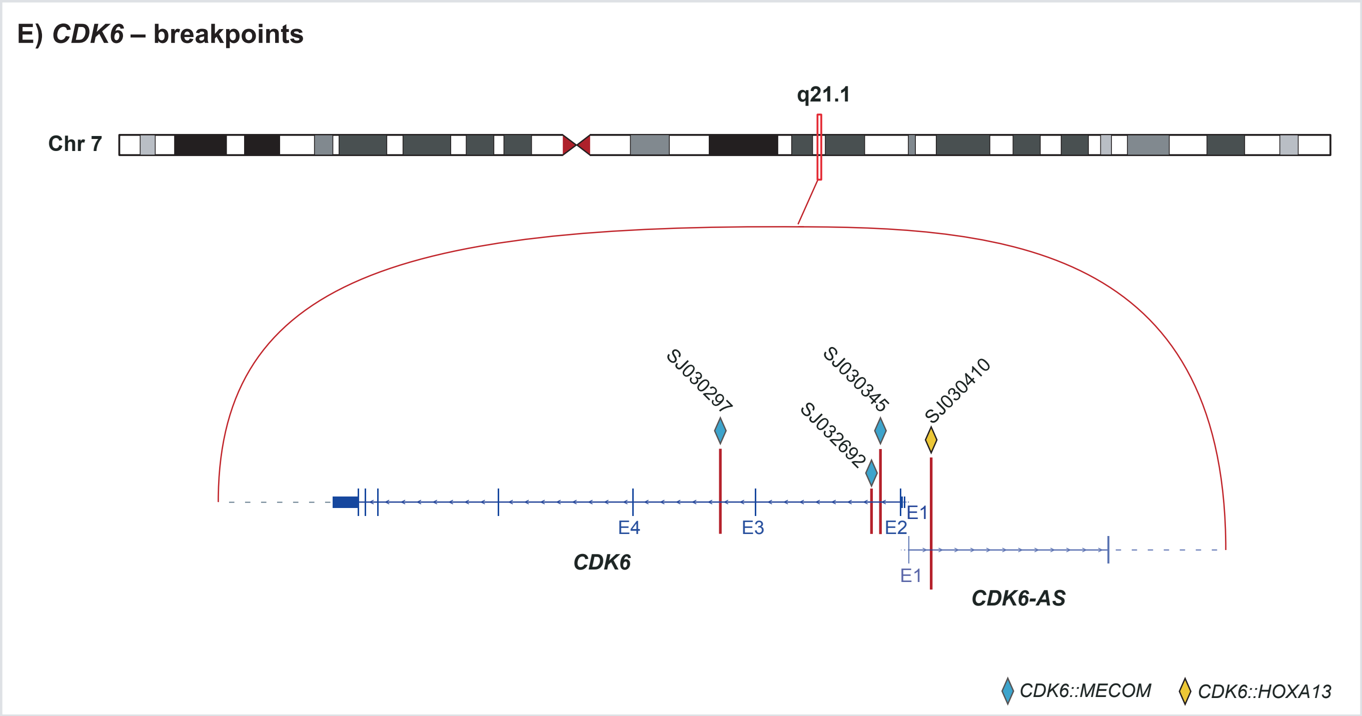

Figure S6. E) Breakpoints in genomic DNA affecting *CDK6*. Affected region within chromosome 7 is marked, *CDK6* (NM_001259.7) and *CDK6-AS* (NR_110088.1) genes are shown schematically. Each vertical line indicates the breakpoint for a given case, colored diamonds refer to the type of underlying gene fusion. Abbreviations: chr, chromosome; E, exon.

Figure S7. Two cases (SJ030153, SJ030431) with t(5;14)(q35;q32.2) by WGS

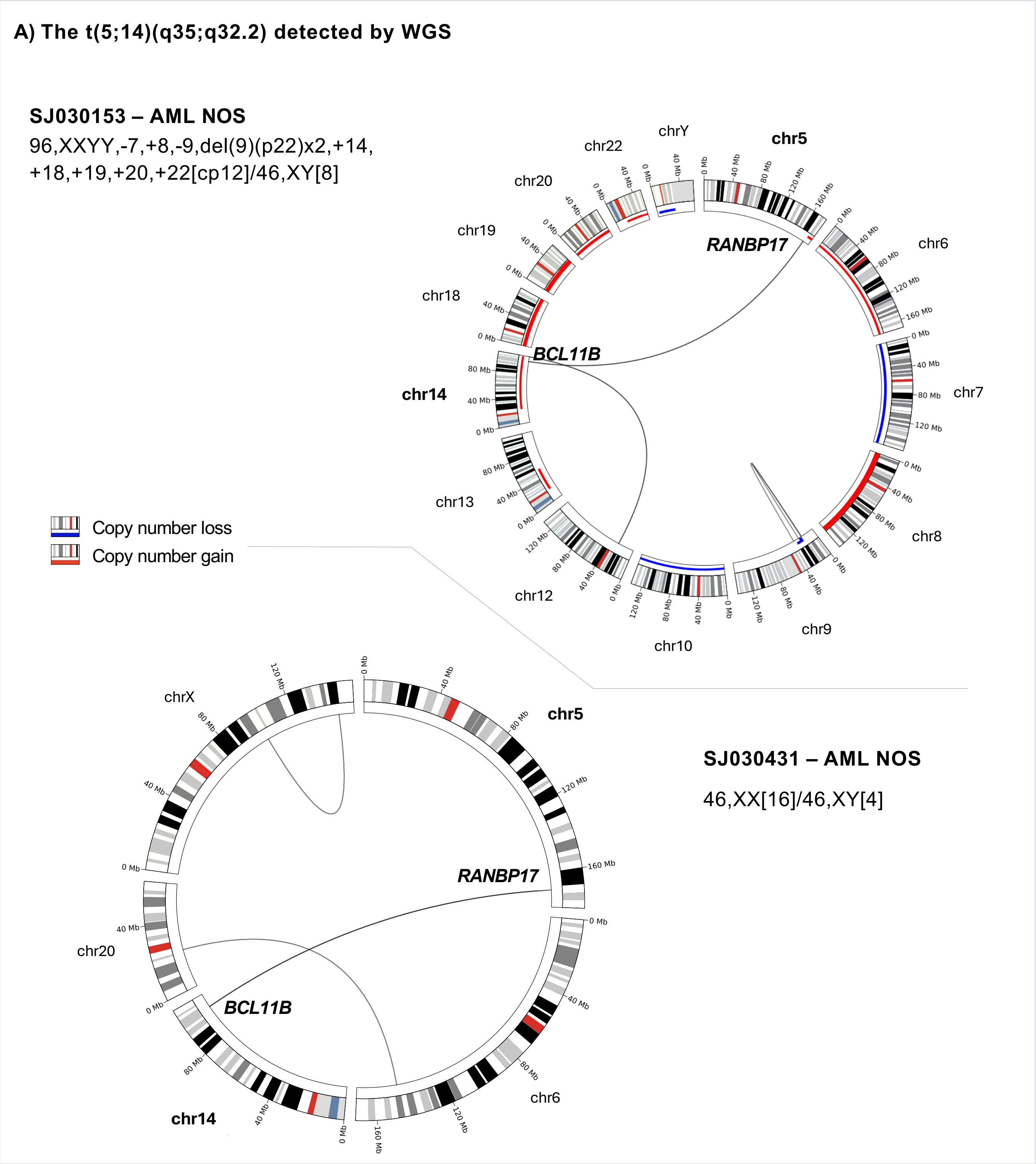

Figure S7. A) Translocation t(5;14)(q35;q32) identified by WGS in two patients shown in CIRCOS plots, suggesting enhancer hijacking fusions involving *BCL11B*, *TLX3* or *NKX2-5*. SJ030153 shows a near tetraploid genome, copy number changes are described in relation to the tetraploid level. Abbreviations: chr, chromosome; NOS, not otherwise specified.

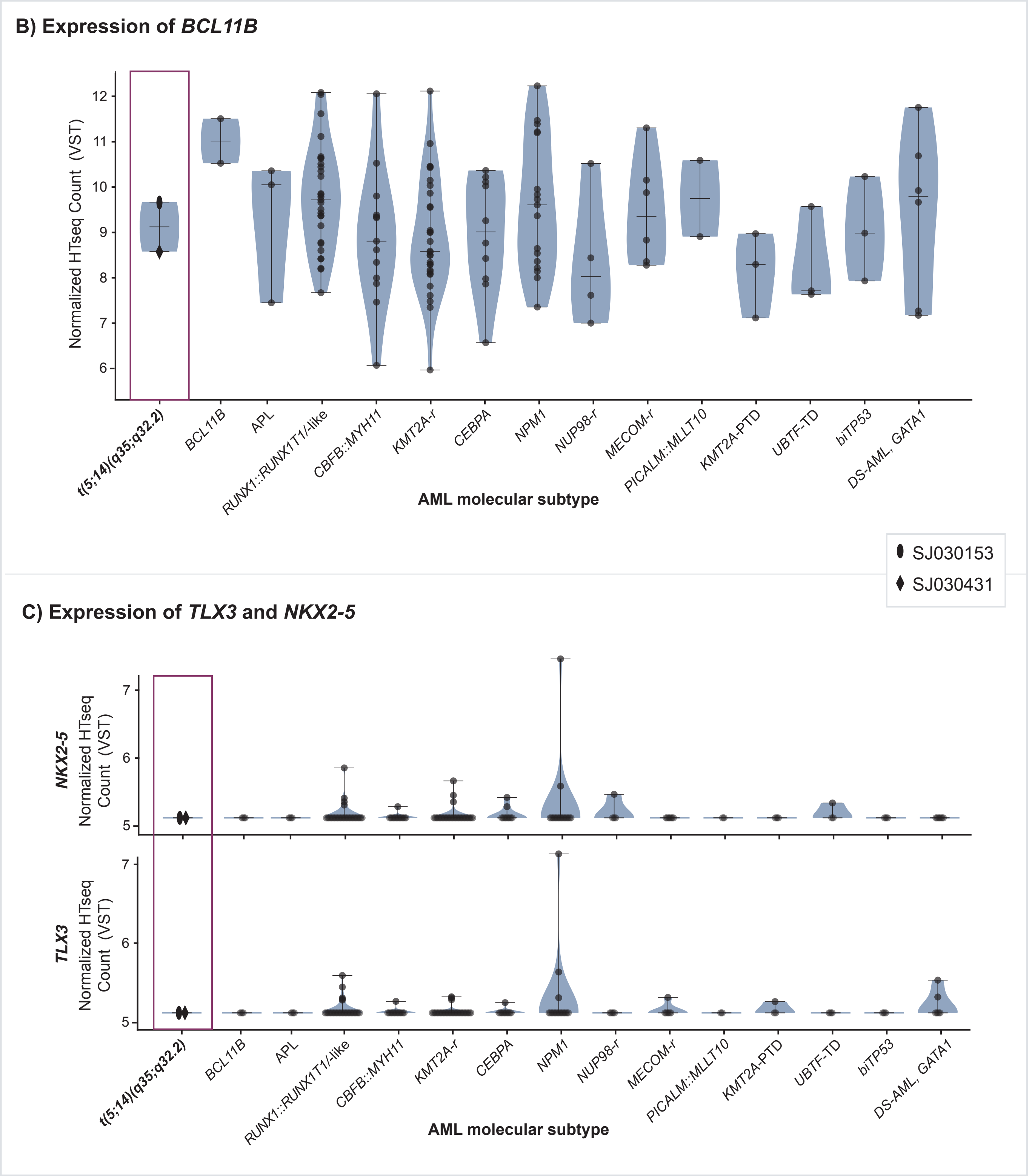

Figure S7. B) and **C) Normalized expression of *BCL11B*, *TLX3* and *NKX2-5*.** Expression values are shown as variance-stabilized counts (VST) derived from HTSeq counts. Normalization aligns with the dispersion-mean relationship, adjusting count data to maintain constant variance across the range of mean expression values. Values are not comparable between genes due to gene-specific dispersion estimates and batch correction (see Supplemen-tary Methods). In this dataset, a baseline of 5.1 (*TLX3, NKX2-5*) and 5.9 (*BCL11B*) corresponds to a raw HTSeq count of 0. AML subtypes

from the study cohort with two or more cases are shown for comparison. *BCL11B*-AML cases, included as a positive control, were obtained from the St. Jude Cloud (https://www.stjude.cloud/). Abbreviations: APL, acute promyelocytic leukemia bi*TP53*, biallelic *TP53* alterations; *CEBPA,* bi*CEBPA*/smbZIP-*CEBPA*; DS-AML, Down Syndrome related AML; PTD, partial tandem duplication; TD, tandem duplication.

Figure S8. Case SJ031206 with *HSPA8::PRDM16* fusion

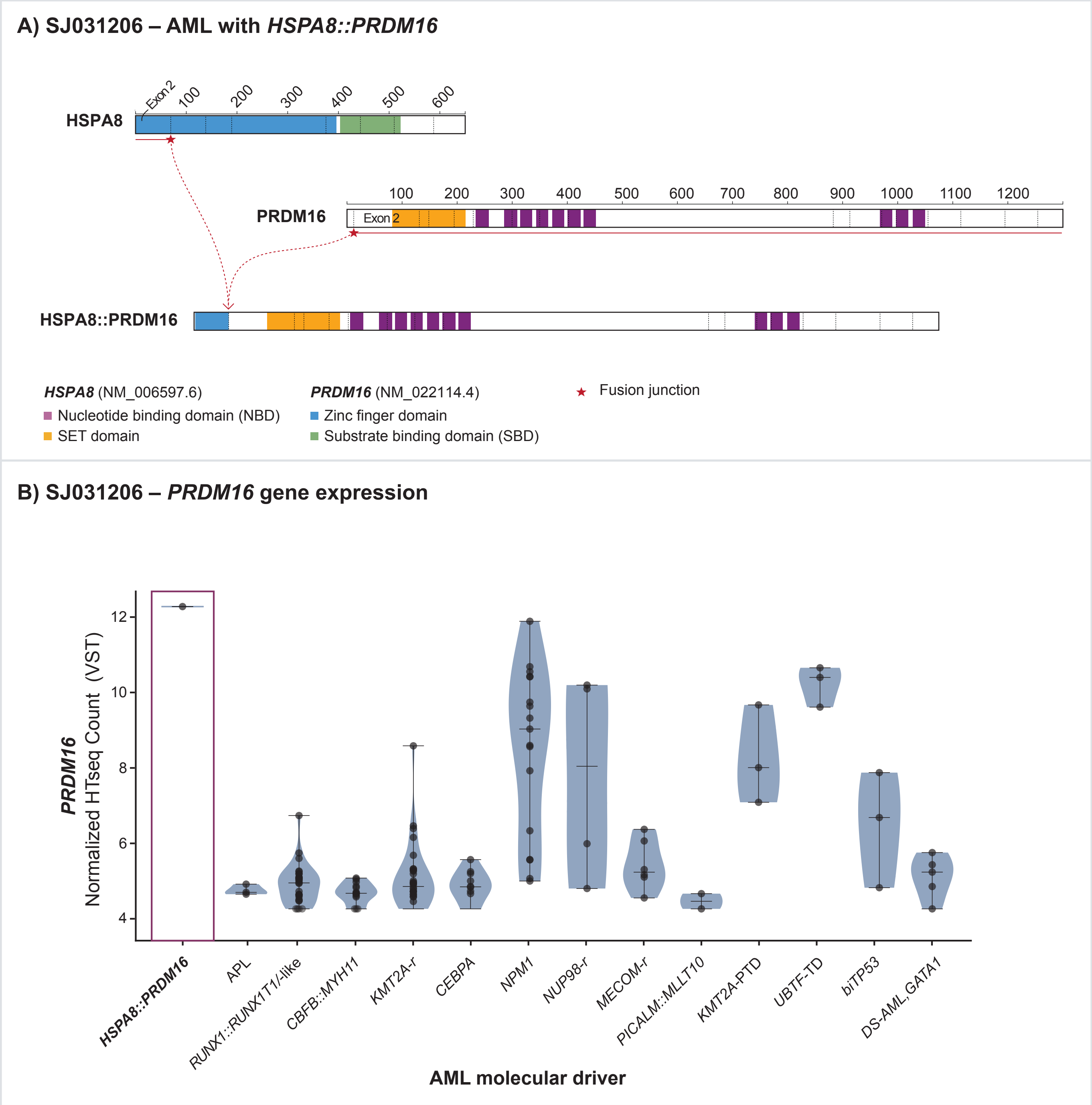

Figure S8. A) WGS identified a balanced translocation t(1;11), with breakpoints in exon 3 of *HSPA8* and intron 1 of *PRDM16*. WTS analysis revealed a complex array of splicing events resulting from this rearrangement. Notably, one of the resulting transcripts encodes an in-frame *HSPA8::PRDM16* fusion with *HSPA8* exon 2 fused to *PRDM16* exon 2, as shown above. Other chimeric transcripts are predicted to be out-of-frame. **B) Normalized expression of *PRDM16*.** SJ031206 is shown on the left along with other AML subtypes from this cohort with two or more cases. *PRDM16* expression values are shown as variance-stabilized counts (VST) derived from HTSeq counts. In this dataset, a baseline of 4.3 corresponds to a raw HTSeq count of 0. **Abbreviations:** APL, acute promyelocytic leukemia; bi*TP53* , biallelic *TP53* alterations; DS-AML, Down Syndrome; PTD, partial tandem duplication; TD, tandem duplication.

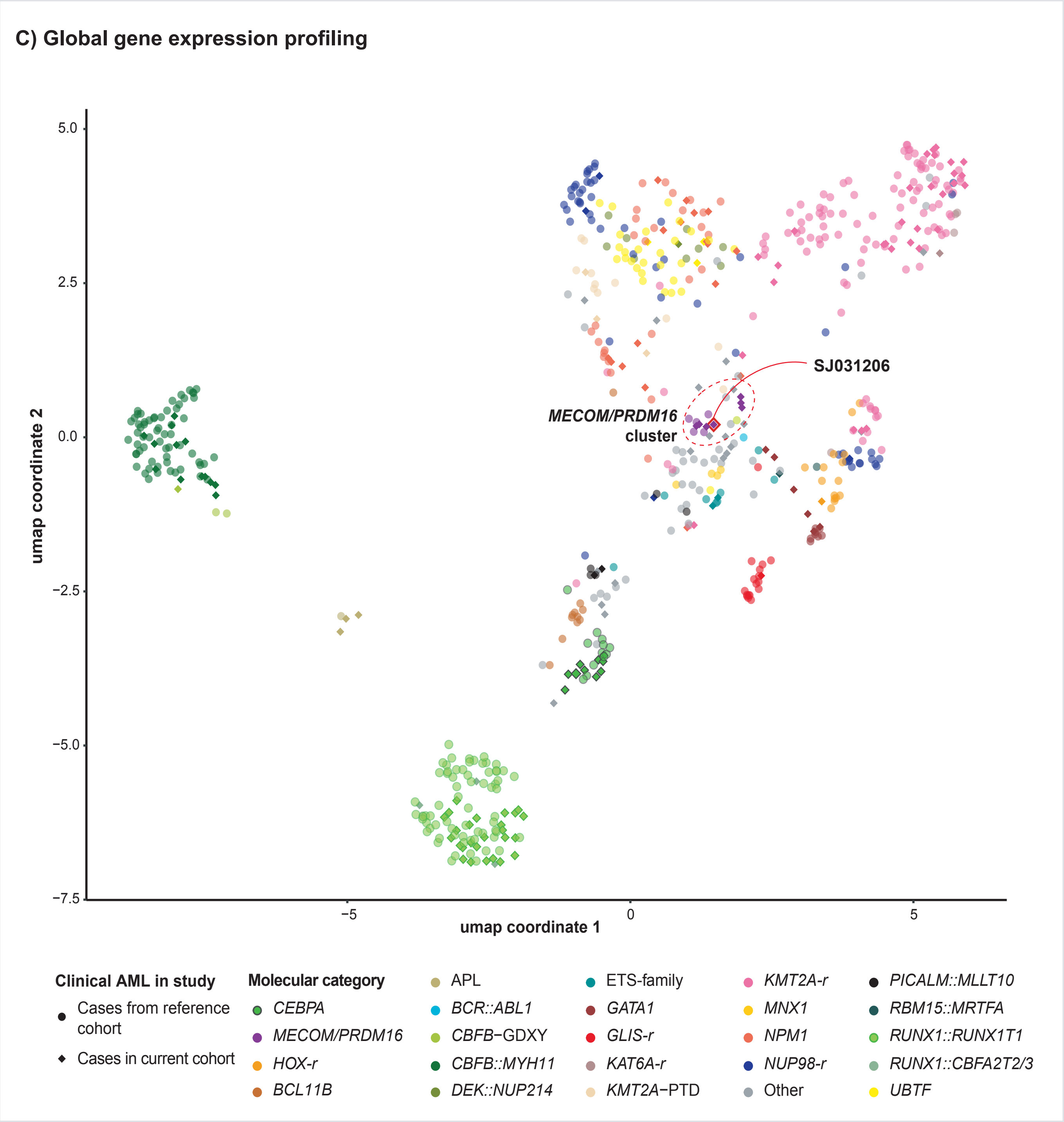

Figure S8. C) Global gene expression profiling by Uniform Manifold Approximation and Projection (UMAP) is shown for an extended cohort of 419 AML patients including 153 AML patients of this study cohort (indicated by diamonds) and 266 cases of a reference cohort generated from data available through St. Jude Cloud. SJ031206 is highlighted and lies within the *MECOM/PRDM16* cluster.

Figure S9. Global gene expression profiling highlighting bi*CEBPA*/smbZIP-*CEBPA* AML molecular subtype

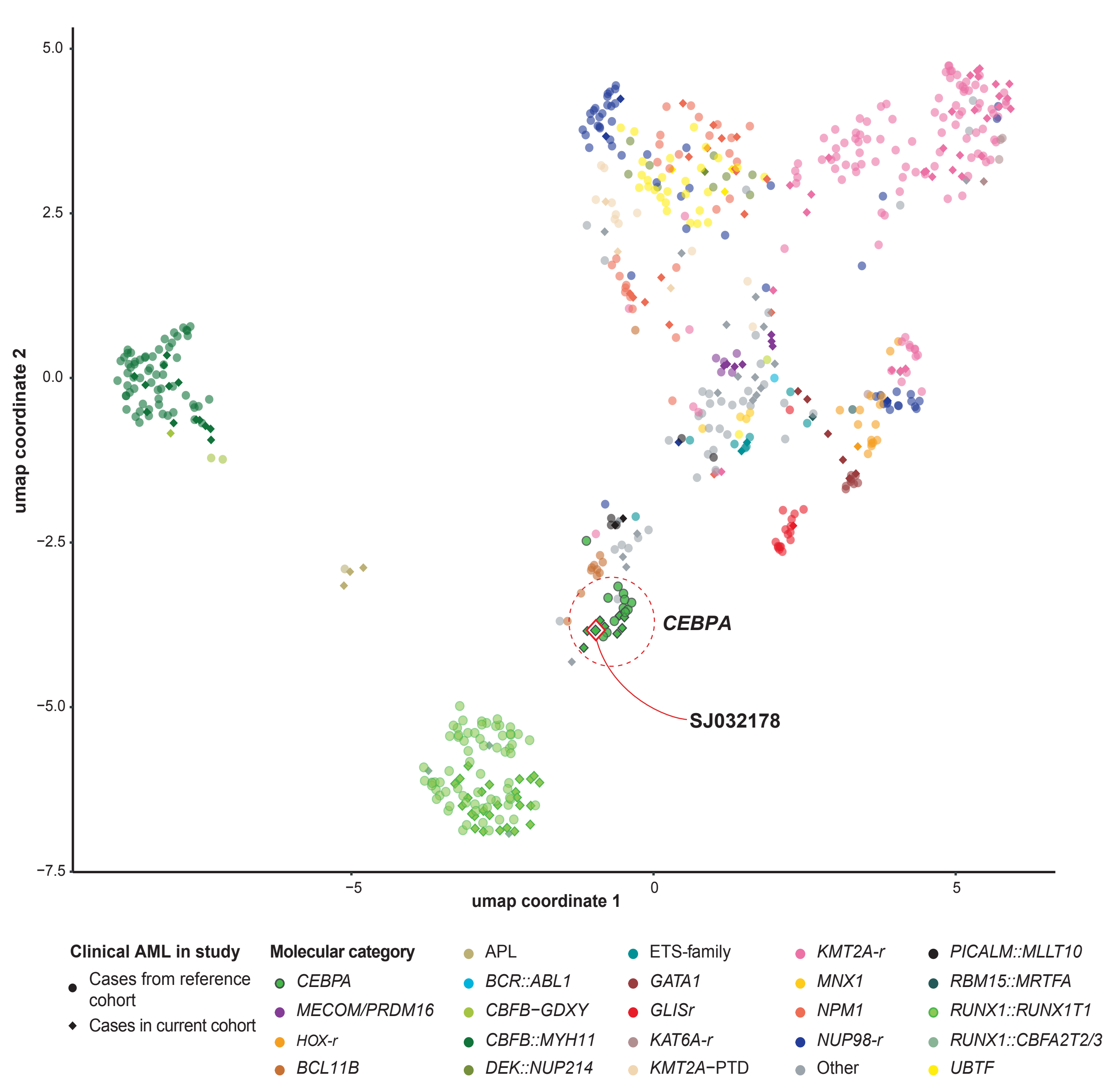

Figure S9. Global gene expression profiling using UMAP shows that case SJ032178, harboring a heterozygous variant in the 5' region of *CEBPA*, clusters with bi*CEBPA*/smbZIP-*CEBPA* AML cases. Abbreviations: UMAP, Uniform Manifold Approximation and Projection.

Figure S10. Global gene expression profiling of cases with *RUNX1*-rearrangements

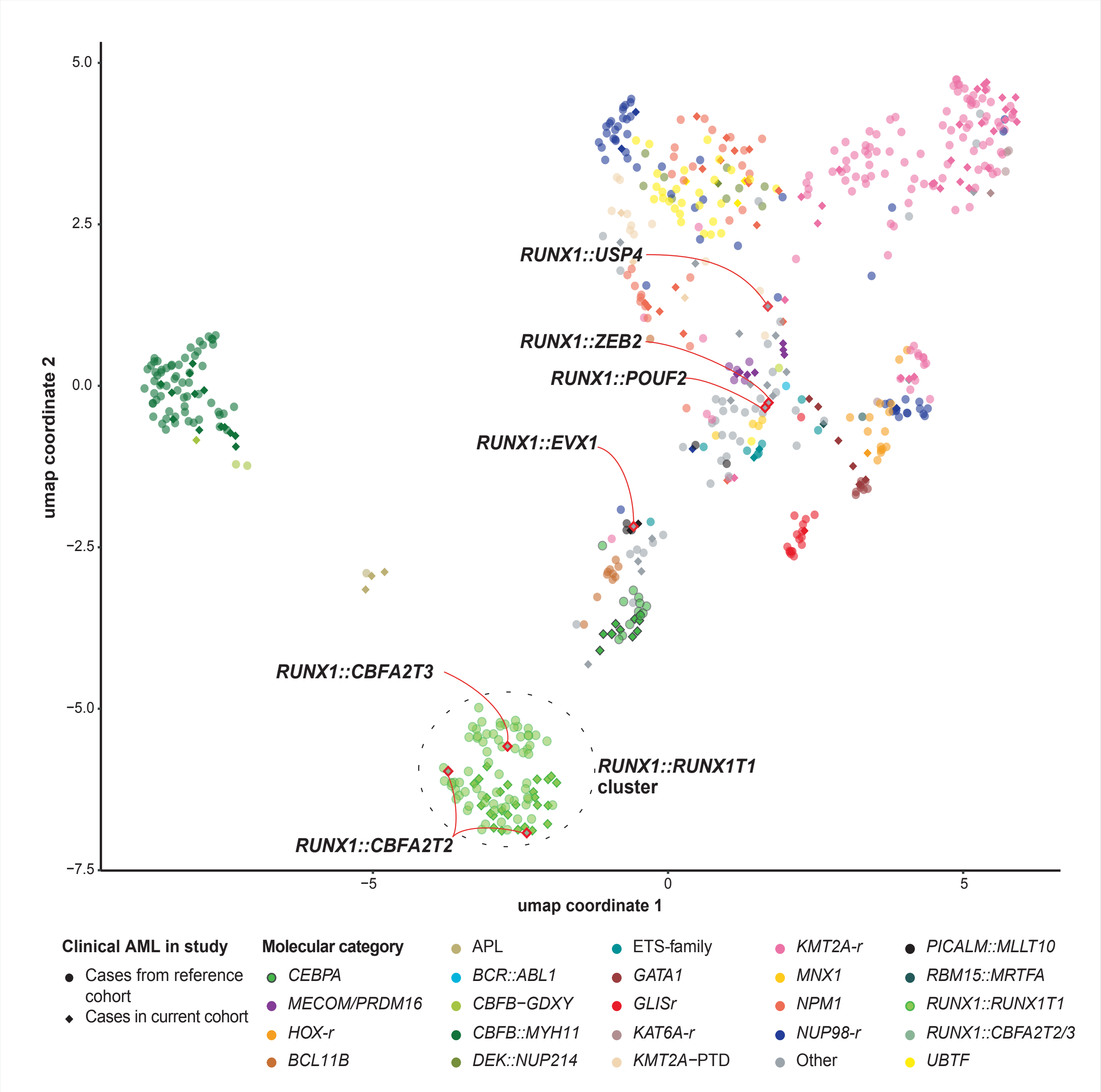

Figure S10. In this study cohort, seven cases harbored in-frame fusions involving 5' exons of *RUNX1* fused to partner genes other than *RUNX1T1*. Global gene expression profiling clustered three *RUNX1::CBFA2T3/2* fusion cases with the *RUNX1::RUNX1T1* group, which is in concordance with other studies considering *RUNX1::CBFA2T3/2* as a form of *RUNX1::RUNX1T1*-like AML (see ref. 26). In contrast, four cases with *RUNX1* fused to other genes (*EVX1*, *POU2F2*, *USP42* and *ZEB2*) neither cluster with the *RUNX1::RUNX1T1* group, nor form a separate cluster in gene expression profiling. This pattern suggests that each rare *RUNX1* fusion may drive leukemogenesis through distinct biological mechanisms, and their clinical significance remains unclear. Collectively, these findings highlight the added value of comprehensive gene-expression profiling in routine molecular diagnostics.

Figure S11. Example cases demonstrating complex genomes resolved by integrated WGS and WTS (iWGS-WTS)

A) SJ030209 – AML with *RBM15::MRTFA*

46,XY,add(1)(p13),?t(3;22)(p21;q13),?ins(5;1)(p15;p34p13)[15]/46,XY[5]

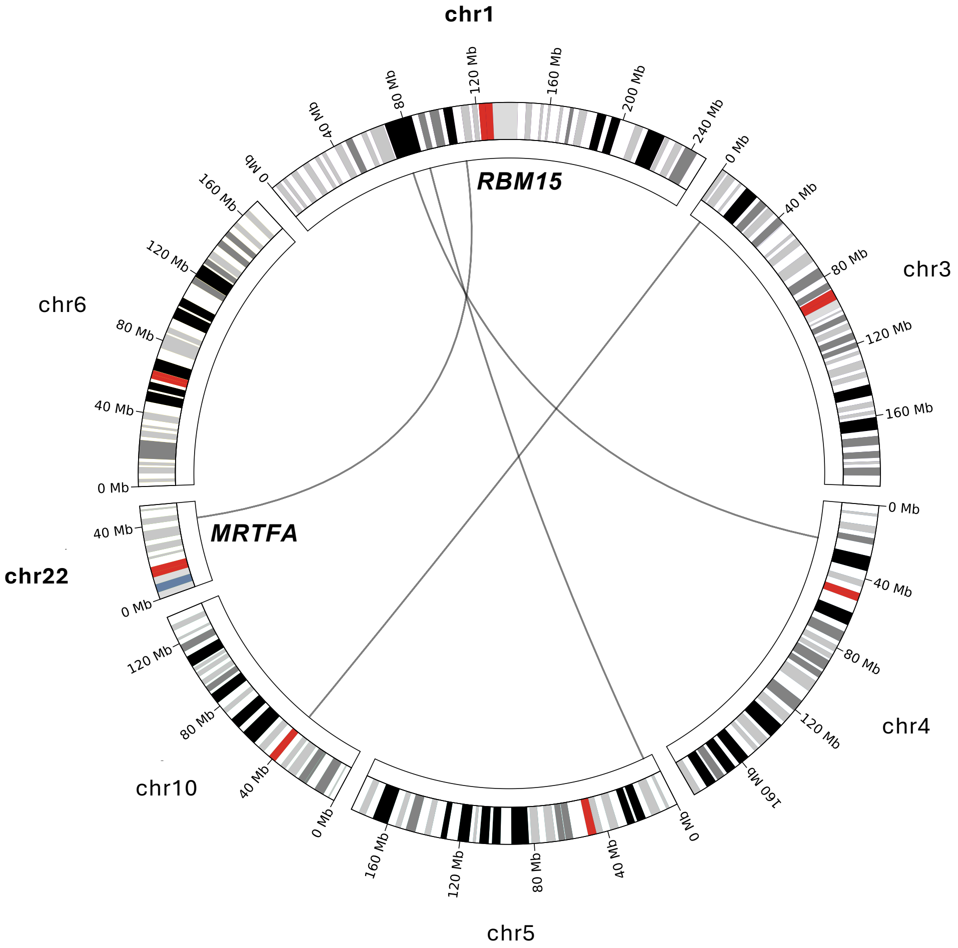

Figure S11. A) SJ030209. WGS revealed multiple balanced chromosome translocations across the tumor genome and elucidated the complex karyotype reported by conventional cytogenetics. In this complex genome, the integrated WGS and WTS (iWGS-WTS) analysis identified an AML driver, *RBM15::MRTFA* fusion. Abbreviations: chr, chromosome; WGS, whole genome sequencing; WTS, whole transcriptome sequencing.

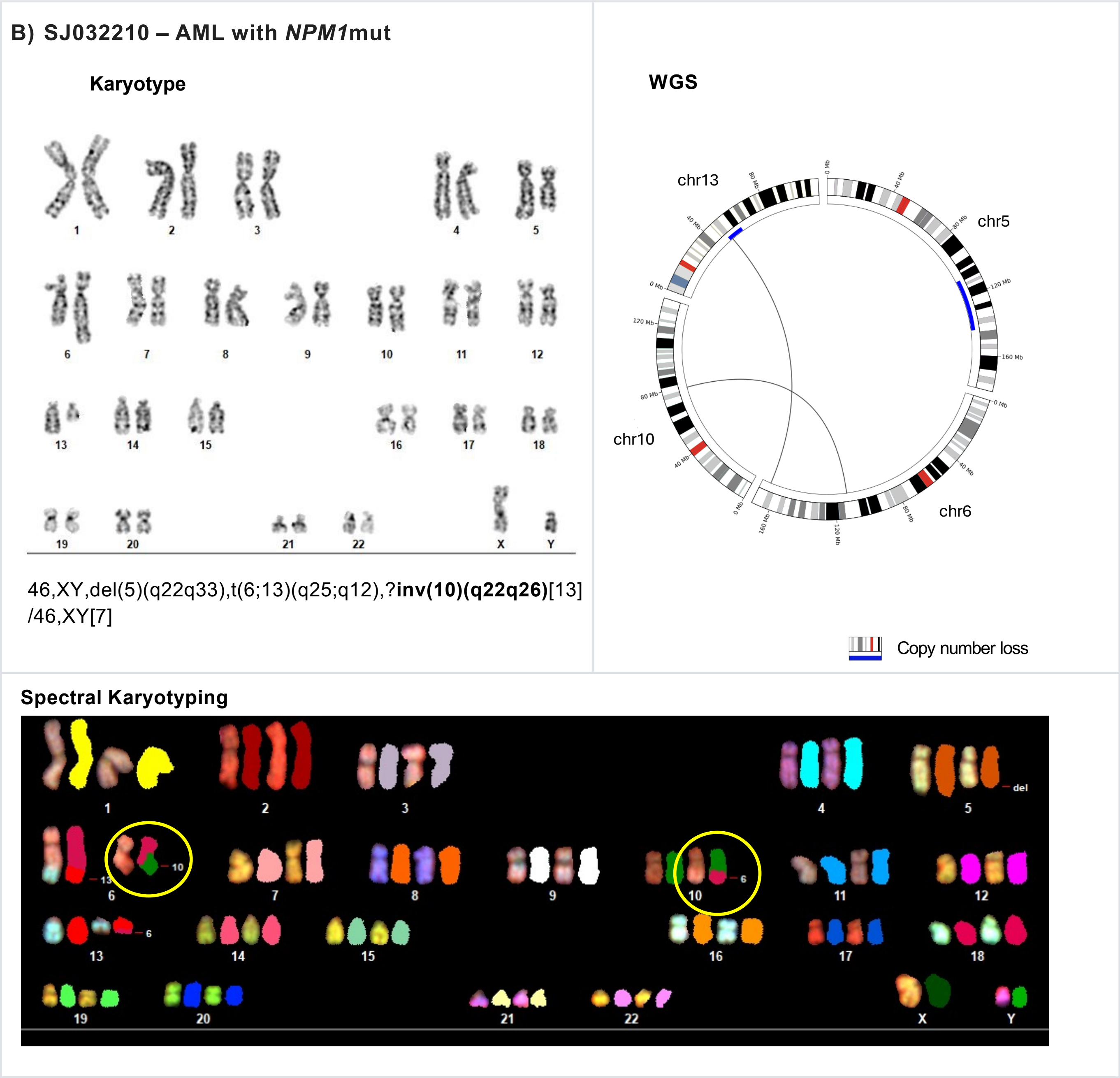

Figure S11. B) SJ032210. A possible inv(10)(q22q26) was observed by G-banded karyotyping. Whole genome sequencing refined this as a t(6;10)(q21;q22.3), SKY (spectral karyotyping) verified the translocation between 6q and 10q. The iWGS-WTS analysis further suggested that the chromosome translocation t(6;10)(q21;q22.3) does not result in a fusion oncogene or disrupt a tumor suppressor gene (data not shown). Instead, an AML-defining *NPM1* mutation was identified (Supplementary Table S2). Abbreviations: chr, chromosome; WGS, whole genome sequencing; WTS, whole transcriptome sequencing.

Figure S12. Overview of the comparison of whole genome sequencing (WGS) and conventional cytogenetics in the detection of large-scale copy number variants (CNVs)

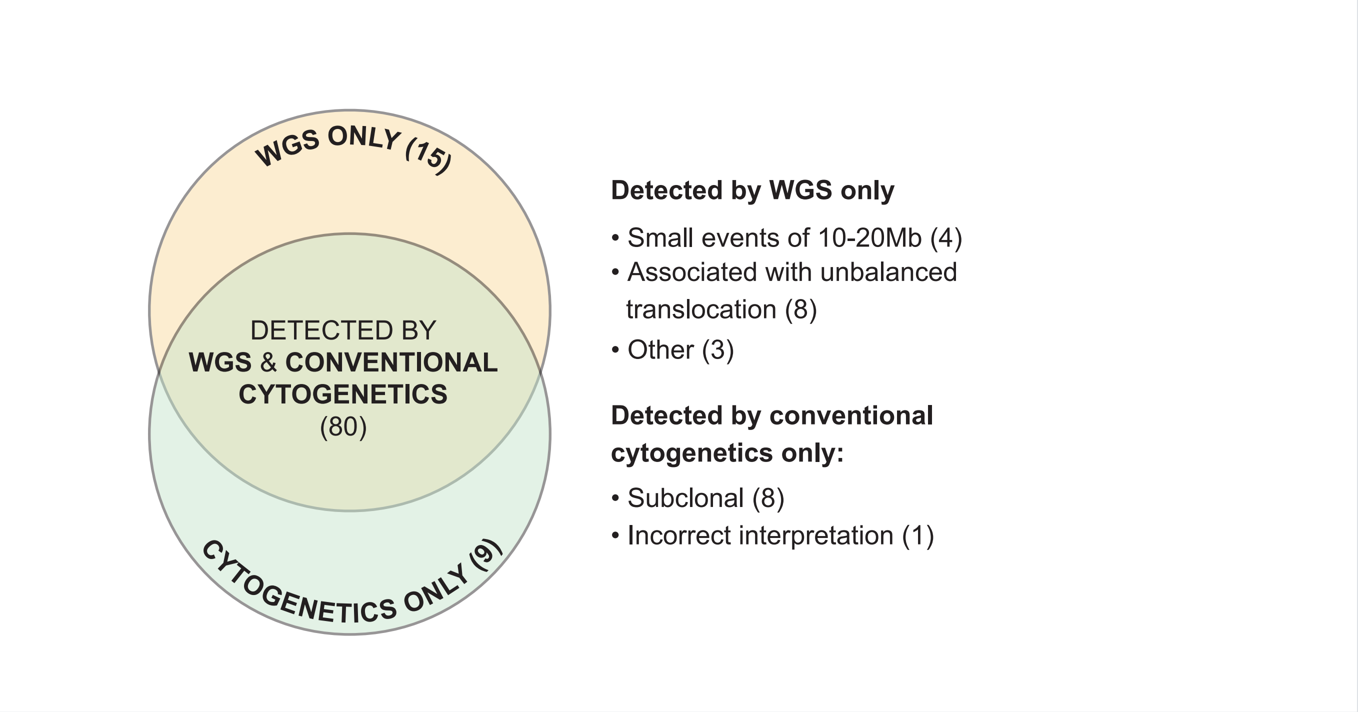

Figure S12. Groups of events detected by cytogenetics or WGS alone are listed on the right. Abbreviations: CNV, copy number variant; Mb, mega base; WGS, whole genome sequencing; WTS, whole transcriptome sequencing.

Figure S13. Abnormal chromosomes observed by conventional karyotype analysis further clarified by Whole Genome Sequencing (WGS)

A) SJ031527 – DS-AML with *GATA1* mut

47,XY,+21c,add(21)(q22.3)[13]/47,XY,+21c[7]

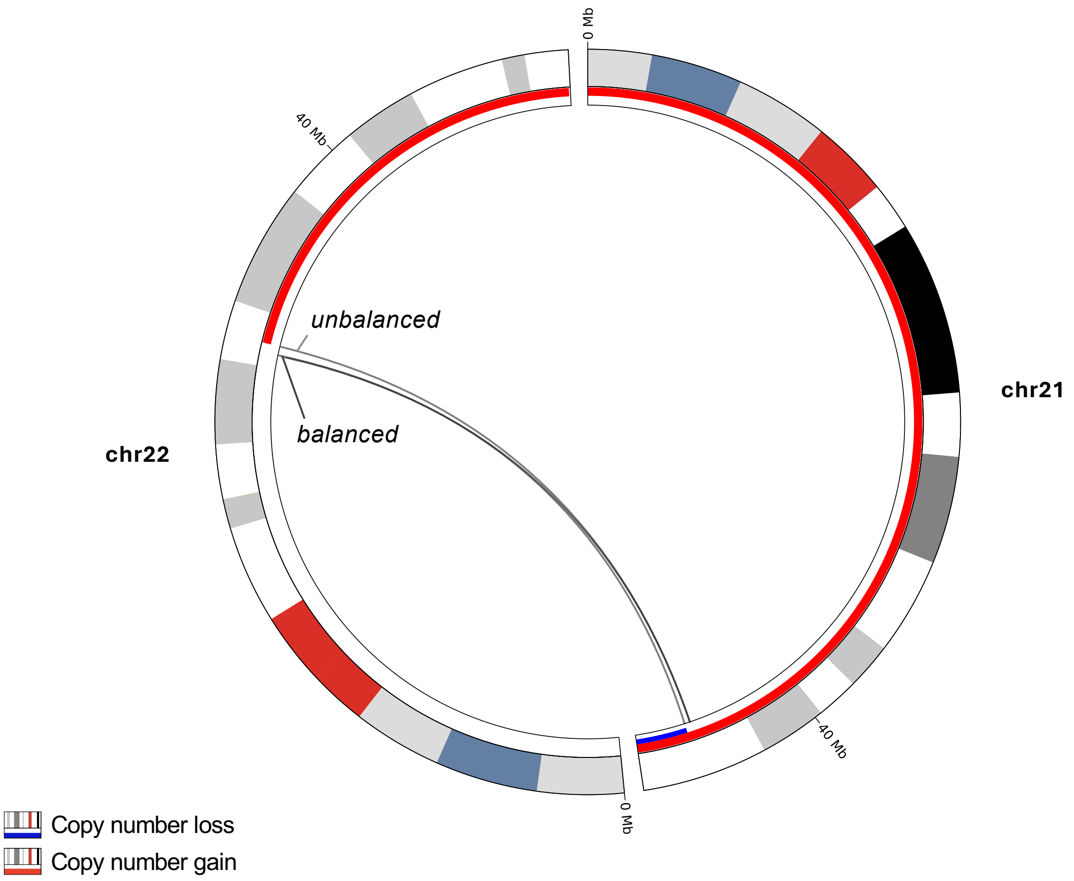

Figure S13. CIRCOS plots of five patients, SJ031527, SJ031601, SJ031719, SJ032364 and SJ032526. Copy number variants and structural variants detected by WGS are shown for affected chromosomes. Outer ring represents chromosomes (chr), bars within represent cytobands (centromere in red). Inner ring shows copy number alterations, loss in blue and gain in red. Lines represent inter- or intra- chromosomal rearrangements. **A) SJ031527**, a case with constitutional trisomy 21 and mutation in *GATA1* (Supplementary Table S2). The add(21)(q22.3) observed by karyotype analysis was further defined by WGS as a der(21)t(21;22)(q22.3;q12.2).

B) SJ031601 – AML with *RUNX1::RUNX1T1*

46,XY,del(7)(q11.1),t(8;21)(q22;q22)[12]/46,XY[1]

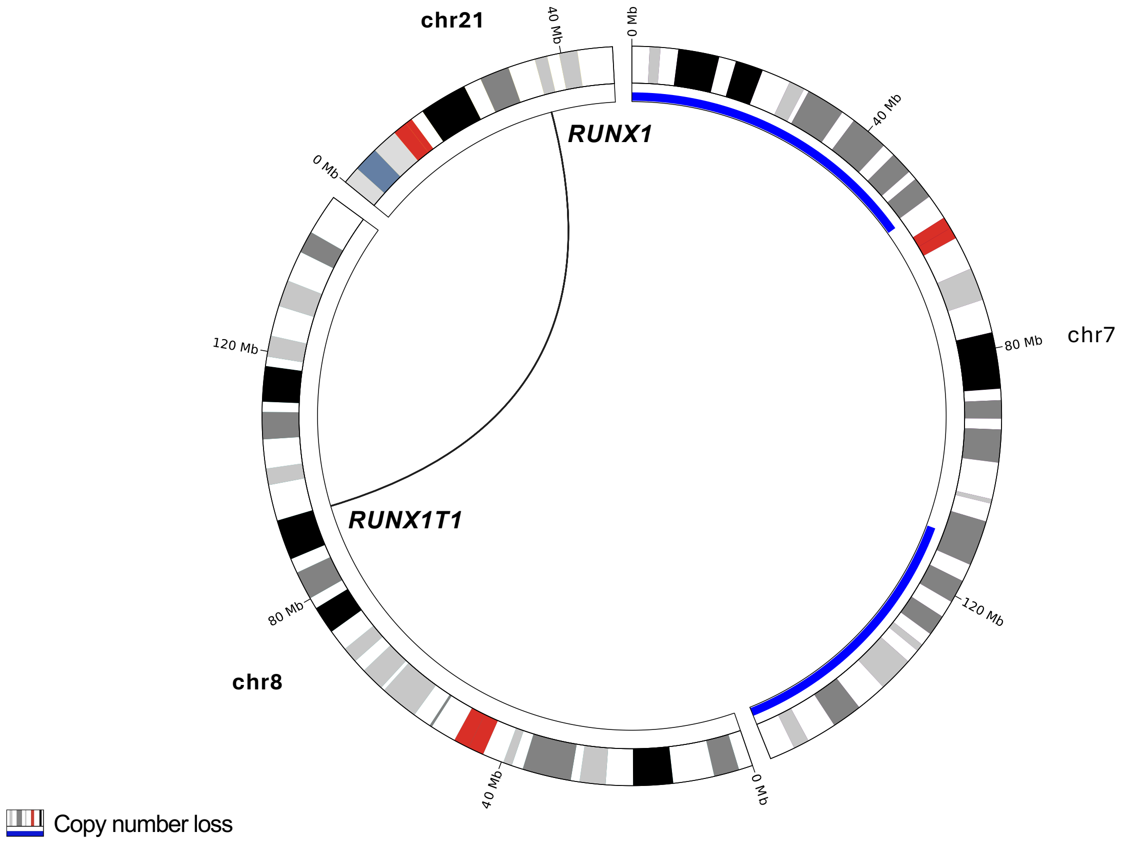

Figure S13. B) SJ031601, **AML with *RUNX1::RUNX1T1***. A del(7)(q11.1) observed by karyotype analysis was further defined by WGS as der(7)del(7)(p11.2)del(7)(q31.1).

C) SJ031719 – AML with *TBL1XR1::RARB*

46,XX,t(3;3)(p24;q26.2),-16,+mar[13]/46,idem,del(11)(q21q23)[3]

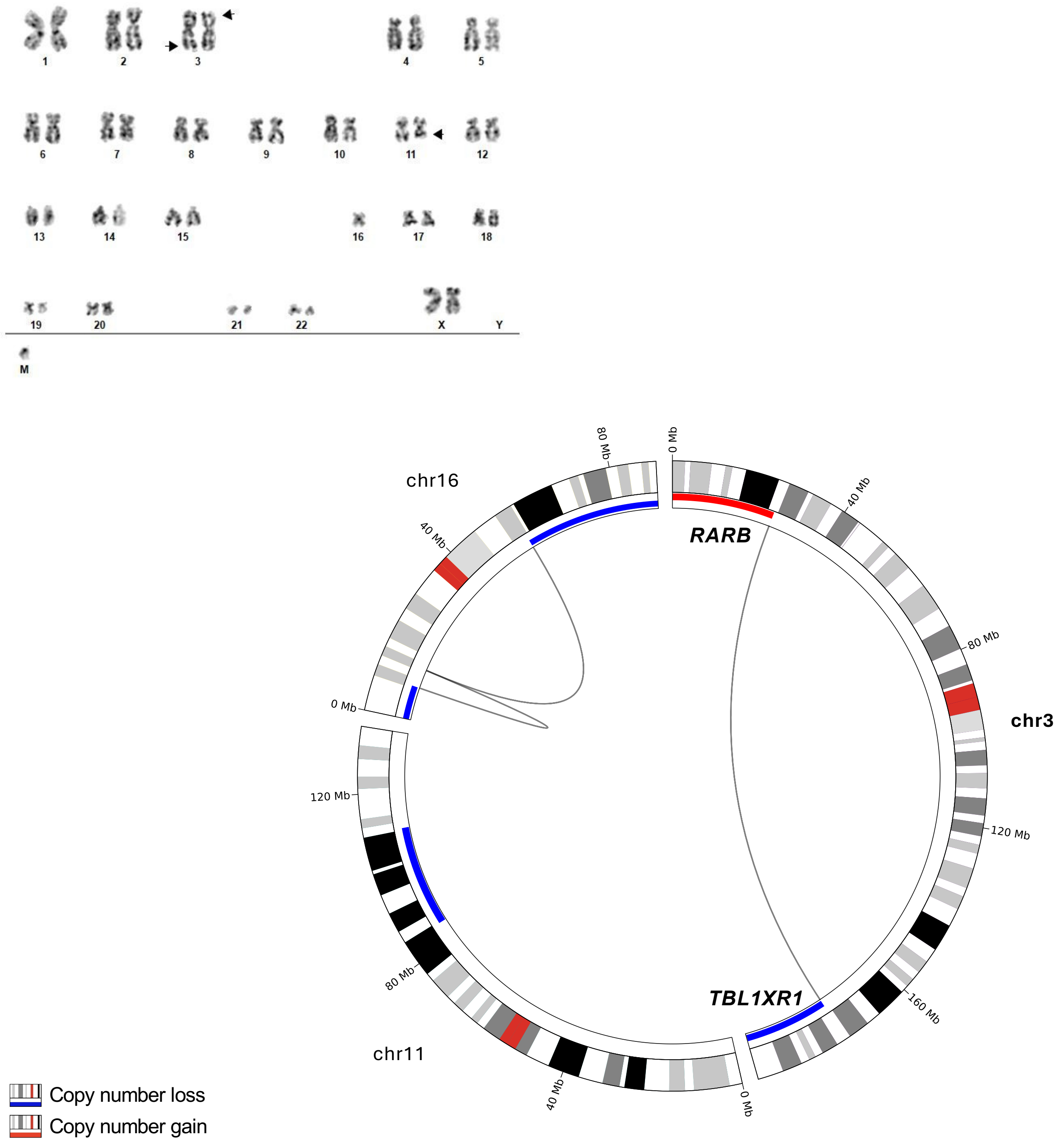

Figure S13. C) SJ031719, AML with *TBL1XR1::RARB*. WGS shows segmental CNVs on 3p and 3q that are associated with the fusion. In addition, deletions in both p and q arms of chr 16 were detected, suggesting the marker chromosome in karyotype was derived from chr 16.

D) SJ032364 – AML with *RUNX1::ZEB2*

48,XX,t(2;11)(q21;q22),+6,+7[11]/48,idem,-7,add(12)(p13),+22[4]/46,XX[5]

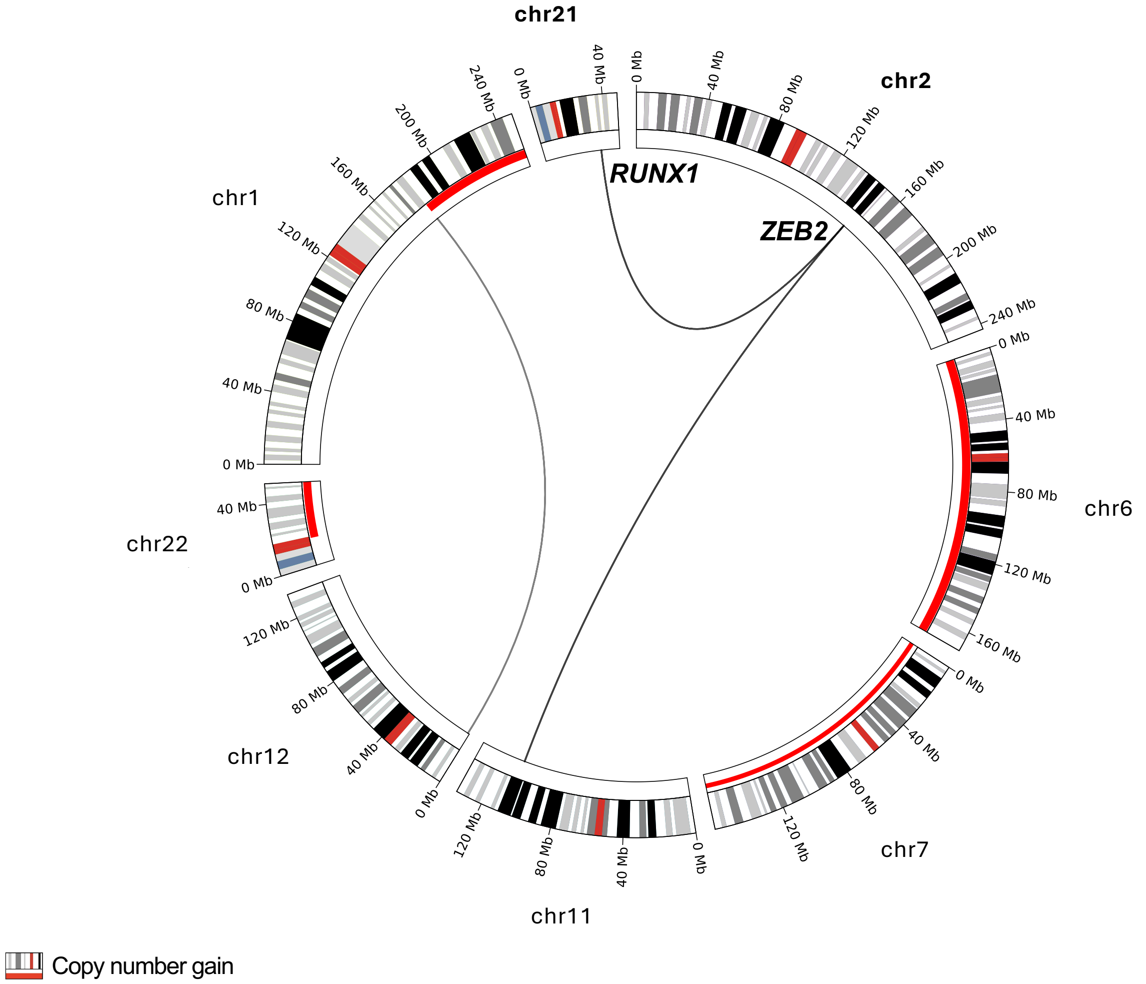

Figure S13. D) SJ032364, AML with *RUNX1::ZEB2*. An add(12)(p13) observed in karyotype is further defined by WGS as der(12)t(1;12)(p13.3;q25.3).

E) SJ032526 – AML NOS

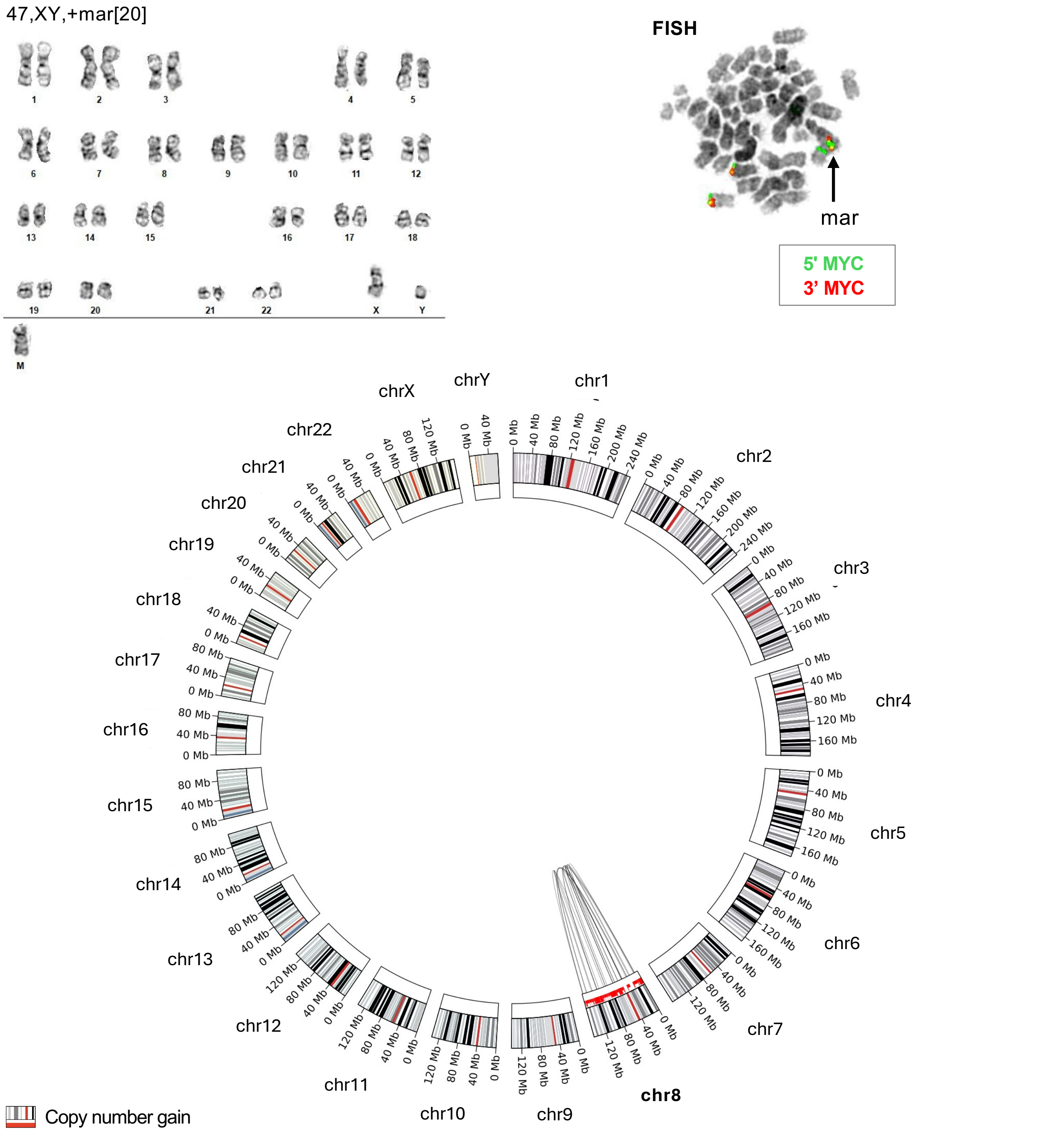

Figure S13. E) SJ032526, AML NOS. WGS revealed copy gains of chromosome 8 and chromothripsis-like changes across chromosome 8, suggesting that the marker chromosome observed in karyotype was derived from chromosome 8. This was further supported by fluorescense in situ hybridization (FISH) using probes targeting *MYC* (8q24).

Figure S14. Detection sensitivity of large scale copy number alterations in dilution series

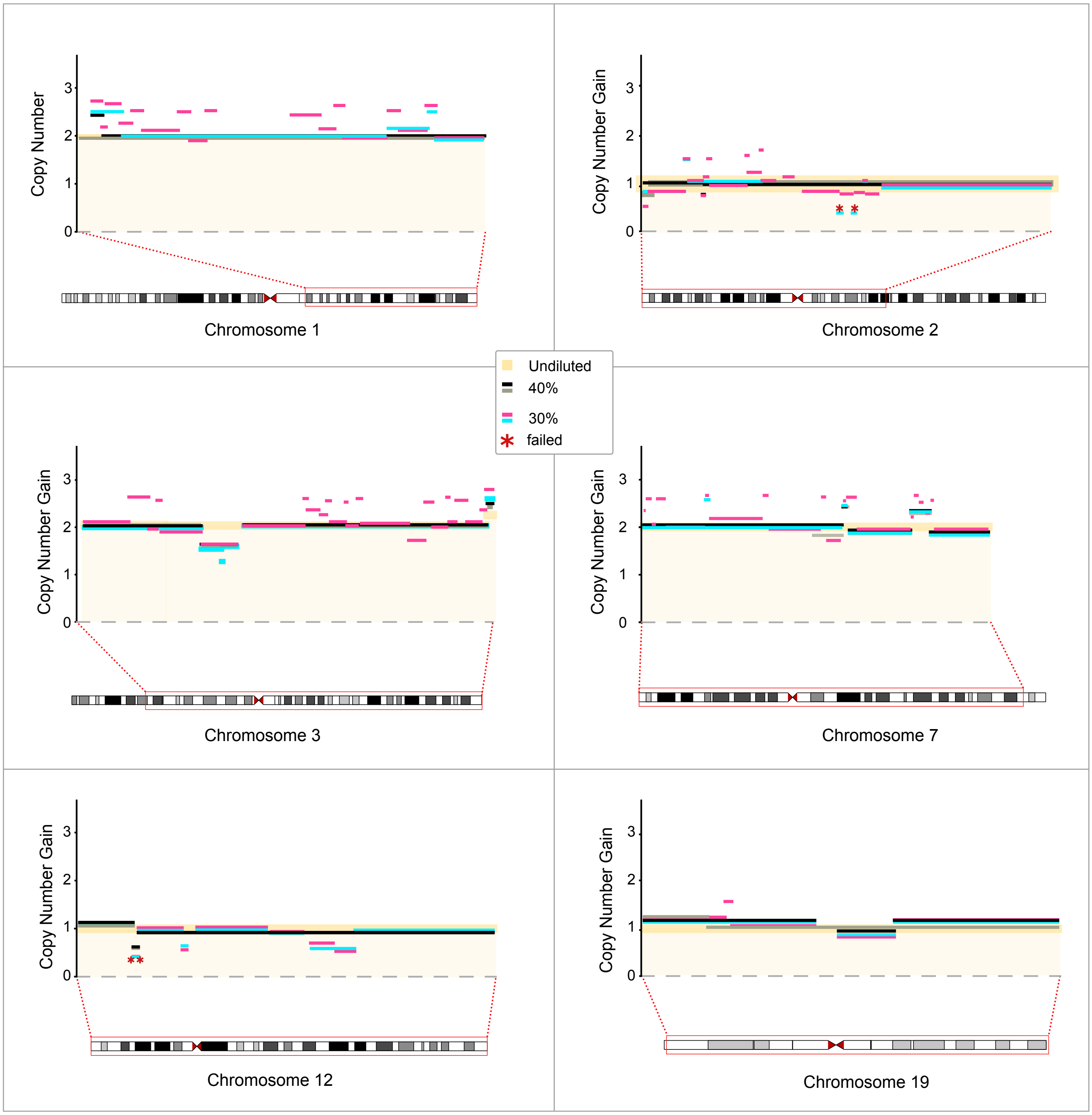

Figure S14. Representative large scale copy number gains detected by WGS are shown for COLO829 and 40% and 30% dilutions (COLO 829 diluted with COLO 829BL), respectively. Regions of copy number gain are indicated schematically on chromosomes depicted below each graph. X-axis represent segment of respective gain, y-axis indicates absolute copy number gain as detected by whole genome sequencing. Results from undiluted COLO 829 samples are depicted in faded yellow, duplicates of sample diluted to 40% original tumor content is shown in black and gray; duplicates of sample diluted to 30% original tumor content are shown in pink and blue. Red asterisk indicates that the respective segment failed the previously established criteria for calling a copy number change.

Figure S15. Comparison of iWGS-WTS, cytogenetics and NGS panel testing in the molecular diagnosis and classification of AML

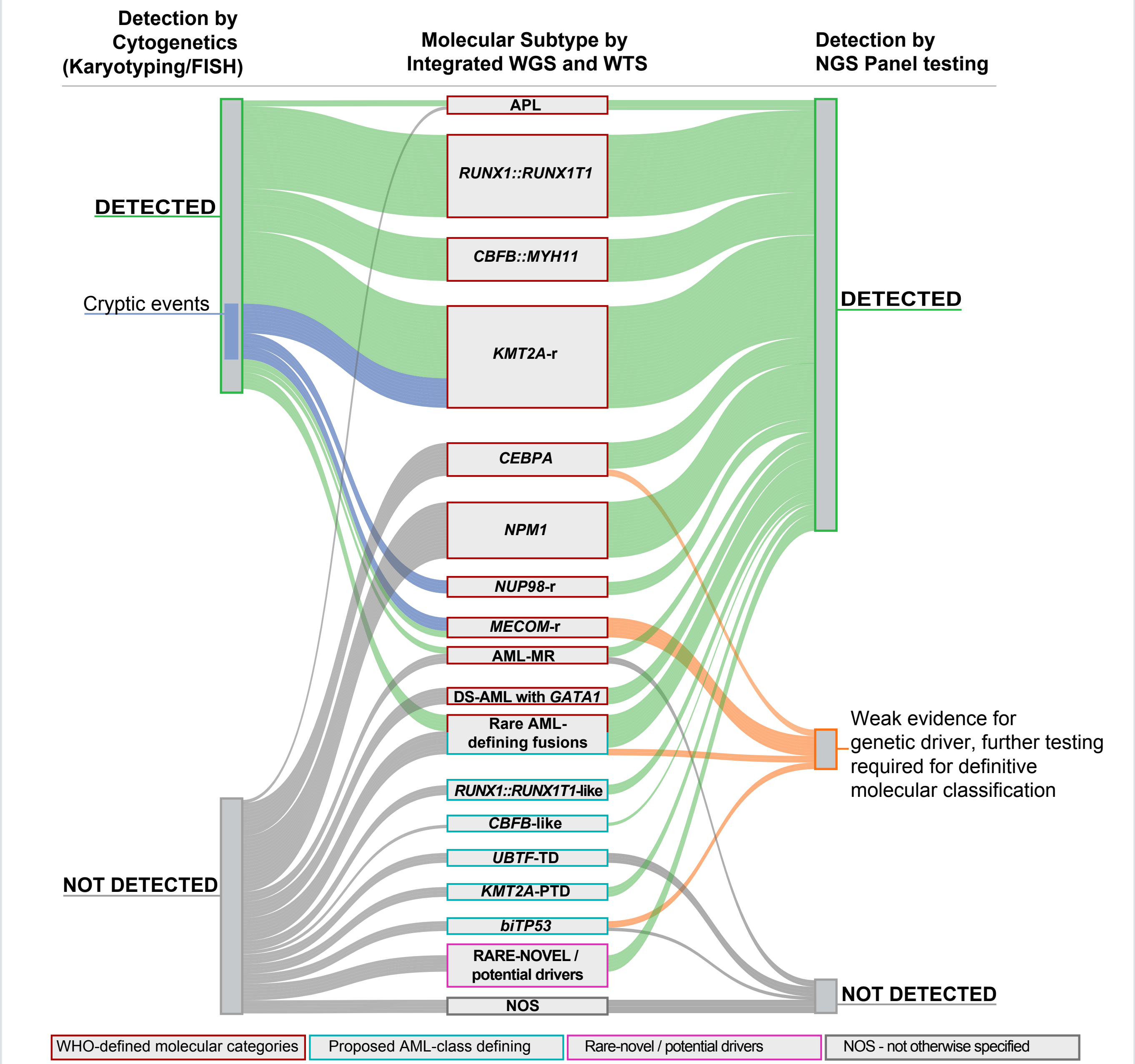

Summary of molecular subtypes identified by different diagnostic methods

	iWGS-WTS	Cytogenetics (Karyotyping/FISH)	NGS Panel testing	Cytogenetics & NGS Panel testing
**Sample requirement**	1x ~ 3mL BMA	1x ~ 3mL BMA	1x ~ 3mL BMA	2x ~ 3mL BMA
**Detection rate – all**	97.4%	57.5%	85.6%	92.8%
**Detection rate – WHO-defined**	100%	69.7%	91.8%	98.4%

Figure S15. Molecular subtype defined by iWGS-WTS is shown in the center column; detection by cytogenetics is shown on the left and NGS panel testing on the right.

Karyotypically cryptic rearrangements diagnosed by or with the help from FISH assays are highlighted in blue. Detection rate and sample requirements are summarized in the lower panel. Abbreviations: APL, acute promyelocytic leukemia; BMA, bone marrow aspirate; DS-AML, Down-Syndrome related AML; FISH, fluorescence in situ hybridization; iWGS-WTS, integrated WGS and WTS; NGS, next generation sequencing; WGS, whole genome sequencing; WTS, whole transcription sequencing.

Section 3. Supplementary References

1. Velazquez-Villarreal EI, Maheshwari S, Sorenson J, Fiddes IT, Kumar V, Yin Y, et al. Single-cell sequencing of genomic DNA resolves sub-clonal heterogeneity in a melanoma cell line. Commun Biol. 2020 Jun 25;3(1):318.

2. Rusch M, Nakitandwe J, Shurtleff S, Newman S, Zhang Z, Edmonson MN, et al. Clinical cancer genomic profiling by three-platform sequencing of whole genome, whole exome and transcriptome. Nat Commun. 2018 Sep 27;9(1):3962.

3. Richards S, Aziz N, Bale S, Bick D, Das S, Gastier-Foster J, et al. Standards and guidelines for the interpretation of sequence variants: a joint consensus recommendation of the American College of Medical Genetics and Genomics and the Association for Molecular Pathology. Genetics in Medicine. 2015 May;17(5):405–24.

4. https://cspec.genome.network/cspec/ui/svi/.

5. Tavtigian S V., Harrison SM, Boucher KM, Biesecker LG. Fitting a naturally scaled point system to the ACMG/AMP variant classification guidelines. Hum Mutat. 2020 Oct 30;41(10):1734–7.

6. Li MM, Datto M, Duncavage EJ, Kulkarni S, Lindeman NI, Roy S, et al. Standards and Guidelines for the Interpretation and Reporting of Sequence Variants in Cancer: A Joint Consensus Recommendation of the Association for Molecular Pathology, American Society of Clinical Oncology, and College of American Pathologists. J Mol Diagn. 2017 Jan;19(1):4–23.

7. Landrum MJ, Lee JM, Benson M, Brown GR, Chao C, Chitipiralla S, et al. ClinVar: improving access to variant interpretations and supporting evidence. Nucleic Acids Res. 2018 Jan 4;46(D1):D1062–7.

8. McLeod C, Gout AM, Zhou X, Thrasher A, Rahbarinia D, Brady SW, et al. St. Jude Cloud: A Pediatric Cancer Genomic Data-Sharing Ecosystem. Cancer Discov. 2021 May 1;11(5):1082–99.

9. Anders S, Pyl PT, Huber W. HTSeq—a Python framework to work with high-throughput sequencing data. Bioinformatics. 2015 Jan 15;31(2):166–9.

10. Love MI, Huber W, Anders S. Moderated estimation of fold change and dispersion for RNA-seq data with DESeq2. Genome Biol. 2014 Dec 5;15(12):550.

11. Leek JT, Johnson WE, Parker HS, Jaffe AE, Storey JD. The <tt>sva</tt> package for removing batch effects and other unwanted variation in high-throughput experiments. Bioinformatics. 2012 Mar 15;28(6):882–3.

12. Umeda M, Ma J, Westover T, Ni Y, Song G, Maciaszek JL, et al. A new genomic framework to categorize pediatric acute myeloid leukemia. Nat Genet. 2024 Feb 11;56(2):281–93.
